# Adding a polyphenol-rich fiber bundle to food impacts the gastrointestinal microbiome and metabolome in dogs

**DOI:** 10.3389/fvets.2022.1039032

**Published:** 2023-01-20

**Authors:** Dale A. Fritsch, Matthew I. Jackson, Susan M. Wernimont, Geoffrey K. Feld, Dayakar V. Badri, John J. Brejda, Chun-Yen Cochrane, Kathy L. Gross

**Affiliations:** ^1^Hill's Pet Nutrition, Inc., Topeka, KS, United States; ^2^Geocyte LLC, Dublin, OH, United States; ^3^Alpha Statistical Consulting Inc., Lincoln, NE, United States

**Keywords:** metabolomics, microbiome, fiber, dogs, post-biotics, polyphenols, pre-biotics

## Abstract

**Introduction:**

Pet foods fortified with fermentable fibers are often indicated for dogs with gastrointestinal conditions to improve gut health through the production of beneficial post-biotics by the pet's microbiome.

**Methods:**

To evaluate the therapeutic underpinnings of pre-biotic fiber enrichment, we compared the fecal microbiome, the fecal metabolome, and the serum metabolome of 39 adult dogs with well-managed chronic gastroenteritis/enteritis (CGE) and healthy matched controls. The foods tested included a test food (TF1) containing a novel pre-biotic fiber bundle, a control food (CF) lacking the fiber bundle, and a commercially available therapeutic food (TF2) indicated for managing fiber-responsive conditions. In this crossover study, all dogs consumed CF for a 4-week wash-in period, were randomized to either TF1 or TF2 and fed for 4 weeks, were fed CF for a 4-week washout period, and then received the other test food for 4 weeks.

**Results:**

Meaningful differences were not observed between the healthy and CGE dogs in response to the pre-biotic fiber bundle relative to CF. Both TF1 and TF2 improved stool scores compared to CF. TF1-fed dogs showed reduced body weight and fecal ash content compared to either CF or TF2, while stools of TF2-fed dogs showed higher pH and lower moisture content vs. TF1. TF1 consumption also resulted in unique fecal and systemic metabolic signatures compared to CF and TF2. TF1-fed dogs showed suppressed signals of fecal bacterial putrefactive metabolism compared to either CF or TF2 and increased saccharolytic signatures compared to TF2. A functional analysis of fecal tryptophan metabolism indicated reductions in fecal kynurenine and indole pathway metabolites with TF1. Among the three foods, TF1 uniquely increased fecal polyphenols and the resulting post-biotics. Compared to CF, consumption of TF1 largely reduced fecal levels of endocannabinoid-like metabolites and sphingolipids while increasing both fecal and circulating polyunsaturated fatty acid profiles, suggesting that TF1 may have modulated gastrointestinal inflammation and motility. Stools of TF1-fed dogs showed reductions in phospholipid profiles, suggesting fiber-dependent changes to colonic mucosal structure.

**Discussion:**

These findings indicate that the use of a specific pre-biotic fiber bundle may be beneficial in healthy dogs and in dogs with CGE.

## 1. Introduction

The gut microbiome is a critical immune and metabolic organ that consists of bacteria, archaea, viruses, and a variety of eukaryotic organisms that are symbiotically associated with the host. Bacteria constitute the largest fraction of these intestinal microorganisms, with metagenomic sequencing demonstrating that >98% of reads from fecal samples assigned to bacteria in both dogs and cats (1–3). A well-functioning gut microbiome modulates the immune system, helps defend against intestinal pathogens, and provides the host with vitamins and nutrients.

Intestinal bacteria coexist in a symbiotic relationship with their mammalian hosts, exerting its influence through conversion of dietary and host-derived substances and molecules into bioactive metabolites known as post-biotics (2). Short-chain fatty acids (SCFAs), metabolites of dietary tryptophan, and secondary bile acids represent examples of post-biotics arising from host-microbiota interactions that influence gut health status, including energetics, permeability, and immunity (4, 5). Gut bacteria ferment undigested fibers and carbohydrates into straight SCFAs that nourish epithelial cells and regulate intestinal motility and cytokine production (5, 6). Certain proteolytic bacteria also generate branched SCFAs and ammonia from excess protein putrefaction (7). The gut microbiota influences tryptophan metabolism into the kynurenine pathway, serotonin pathway, and indole-containing aryl hydrocarbon receptor ligands, directly and indirectly impacting the inflammatory response, immune homeostasis, epithelial function, and gastrointestinal signaling (8, 9).

The impact of dietary fibers on the gastrointestinal health of dogs has been well-established (10). Historically, fibers have been classified according to their solubility, dispersibility in water, and fermentation profiles (11, 12). There are numerous publications regarding the use of fibers to normalize canine stool firmness (13–15); however, research into the impact of different fibers on the microbial processes of proteolysis and saccharolysis and the accompanying production of antioxidants and anti-inflammatory post-biotics is limited (16–18). While many fibers exhibit similarities based on these characteristics (19), not all provide the same health benefits. In fact, physicochemical characteristics, surface area, and hydration properties may be better indicators of the health benefits associated with fibers. The impact of additional properties of fibers, such as the presence of bound bioactive compounds, is also gaining recognition (12, 20). Foodstuffs and compounds that promote the growth of beneficial microbes and serve as substrates for post-biotic metabolism are known as pre-biotics. For example, fruits and vegetables are rich pre-biotic sources of plant fibers and polyphenols (21), which are a group of compounds that include flavonoids, tannins, and phenolic acids and their derivatives (22). Plant-derived resistant starches that bypass digestion in the small intestine and reach the colon intact also represent a source of fiber. Abundance of resistant starch in dry pet foods is roughly inversely proportional to the intensity of extrusion processing, which involves mechanical shear forces and heat (23). The gut microbiota of dogs is capable of fermenting a wide range of plant fibers and metabolizing polyphenol conjugates and oligomers into more bioavailable forms, while the presence of dietary polyphenols can also influence the composition and function of bacterial populations in the gut microbiota (24, 25). Dietary polyphenol supplementation has been proposed as an intervention for acute diarrhea in dogs, due to the antioxidant, anti-inflammatory, and microbiome-sculpting properties of polyphenols and their catabolites (26).

While variations in dietary composition can alter the composition and function of the gut microbiome of dogs (16, 27, 28), the optimal characteristics of dietary fiber in terms of fermentability, physiochemistry, and bound metabolites are under investigation (29). Consequently, a novel pre-biotic fiber bundle that consists of fibers specifically selected for their pre- and post-biotic activity, water-holding, and stool-bulking capacities was developed. Our previous work has demonstrated that dietary interventions with this pre-biotic fiber bundle have improved stool scores, shifted fecal microbial metabolism from deleterious putrefactive to desirable saccharolytic metabolism, and increased fecal anti-inflammatory and antioxidant metabolites compared with control foods in both dogs and cats (16, 17, 30–33). In a study of dogs with chronic large bowel diarrhea, a therapeutic food that included the pre-biotic fiber bundle significantly and rapidly improved stool consistency, resolved clinical signs of chronic diarrhea, and improved stooling behaviors and quality of life (30). The food also shifted the functional capacity of the GI microbiome and its metabolism toward saccharolytic fermentation from proteolytic putrefaction, increased the abundance of bioavailable post-biotics, and modulated inflammatory and colonic mucosal signatures compared to the pre-intervention baseline state (16).

We hypothesize that differences in fiber content and composition in foods for dogs with gastrointestinal disorders may lead to differences in the fecal and systemic impact of these foods, differences that may be mediated by the dogs' microbiome and metabolism. To better understand how microbiome and metabolism interact to impart the therapeutic benefit of the novel pre-biotic fiber bundle and to determine how differences in fibers alter their fecal and systemic impact, we conducted a randomized, controlled crossover study investigating fecal composition, microbiome, and metabolomic signatures in adult dogs consuming a food containing the pre-biotic fiber bundle, a control food lacking the bundle, or another fiber-based therapeutic food for dogs with gastrointestinal disorders.

## 2. Materials and methods

The study was a randomized, crossover design conducted between August and December 2016 that comprised four phases, each of which lasted 28 days (Supplementary material 1). In Phase 1, all dogs received the control food. In Phase 2, dogs were randomized into Group 1 and received Test Food 1 (TF1), which included the pre-biotic fiber bundle, or Group 2 and received another therapeutic food. In Phase 3, all dogs again received the control food (CF). Finally, in Phase 4, dogs received the test food that they did not receive in Phase 2 [Test Food 2 (TF2) in Group 1 and TF1 in Group 2]. All dogs were fed twice daily, once in the morning and again in the afternoon. All study protocols were reviewed and approved by the Institutional Animal Care and Use Committee (CP693a) of Hill's Pet Nutrition, Inc., in Topeka, KS, USA, and all methods were carried out in accordance with relevant institutional and national guidelines and regulations.

### 2.1. Study dogs

This study subject population consisted of adult beagles housed at the Hill's Pet Nutrition Center (HPNC), as previously described (17), with both healthy dogs and those diagnosed with naturally occurring chronic gastroenteritis, diagnosed by the HPNC veterinarian based on attitude/activity, appetite, vomiting, stool consistency, stool frequency, weight loss, albumin levels, and histopathological analysis by endoscopy and microscopic assessment by an independent veterinarian pathologist when it was deemed in the best interest of the dog (e.g., dog not of advanced age). Healthy control dogs were matched to dogs with CGE by age, weight, and gender. Demographics data for study participants are presented in Table 1. Dogs with CGE had been under the care of the HPNC veterinarian for >15 months and their disease was considered well-managed at the time of the trial. This contrasted with the previous study in which the CGE dogs presented with active signs at the start of the trial (17). Dogs were considered healthy when there was no evidence of chronic systemic disease from physical examination, complete blood count, serum biochemical analyses, urinalysis, or fecal examination for parasites; exclusion criteria were recorded instances of gastrointestinal upset (vomiting, diarrhea) or abnormally low appetite. All dogs were pair-housed in spacious indoor rooms with natural light at HPNC and had the opportunity for behavioral enrichment by interacting with each other, as well as through play time with caretakers, daily runs outside, and access to toys. Dogs were fed twice daily and had *ad libitum* access to water. All dogs were immunized against canine distemper, adenovirus, parvovirus, Bordetella, and rabies, were monitored for parasites, and received routine heartworm preventative. Symptoms of CGE dogs were managed with bismuth subsalicylate, prednisolone, cobalamin, and omeprazole as needed to maintain their quality of life.

**Table 1:** Demographic data for dogs enrolled in study.

**Characteristic**	**Group 1**	**Group 2**	***P*-value**
Age, years, mean ± SD	7 ± 3.7	8 ± 3.6	0.47
Weight, kg, mean ± SD	12 ± 2.6	11 ± 2.1	0.42
Sex, *n* (%)		
Females	5 (23)	6 (30)	0.73
Males	17 (77)	14 (70)	

### 2.2. Study foods

The nutrient profiles of the 3 dry foods used in the study are listed in Table 2. The CF and TF1 differ only in that TF1 included the pre-biotic fiber bundle. The pre-biotic fiber bundle in TF1 (Hill's^®^ Prescription Diet^®^ Gastrointestinal Biome dry dog food) includes ground pecan shells, flaxseed, dried beet pulp, dried citrus pulp, pressed cranberries, and psyllium seed husk, while the fiber components of TF2 (Royal Canin^®^ Veterinary Diet Gastrointestinal High Fiber dry dog food) includes powdered cellulose, rice hulls, dried beet pulp, psyllium seed husk, and fructooligosaccharides. In CF, cornstarch was used in lieu of the TF1-containing fiber bundle. TF2 is a commercially available therapeutic product designed to help manage fiber-responsive gastrointestinal conditions with a similar nutrient profile and different functional ingredients. All 3 foods represent complete and balanced dry nutritional pet foods and met 2017 Association of American Feed Control Officials guidelines for maintenance of adult dogs (34). All dogs were offered an appropriate number of calories to maintain body weight, with pre- and post-weights recorded to assess intake. Caloric needs were also calculated, in part, using an activity factor, to ensure that the dogs' individual nutritional needs were met throughout the trial. The nutritional needs, health, and wellbeing of the dogs included in the study were overseen by the veterinarian and animal care technician team at HPNC.

**Table 2:** Compositional analysis for foods administered in study.

**Nutrient**	**CF**	**TF1**	**TF2**
Protein, %	18.1	19.8	23.3
Fat, %	11.6	12.3	18.5
Crude fiber, %	2.5	6.6	10.1
Soluble fiber, %	1.6	2.6	3.7
Insoluble fiber, %	5.7	13.7	18.8
Nitrogen free extract, %	53.3	47.2	31.8
Ash, %	5.6	6.0	8.3
Calcium, %	0.96	1.08	1.04
Magnesium, %	0.07	0.10	0.08
Phosphorus, %	0.67	0.65	0.89
Potassium, %	0.83	0.93	0.74
Sodium, %	0.34	0.37	0.35
Eicosapentaenoate (EPA), %	0.26	0.27	0.17
Docosahexaenoate (DHA), %	0.19	0.21	0.08
Sum *n*−3 fatty acids, %	0.79	1.41	0.51
Sum *n*−6 fatty acids, %	3.11	3.28	3.42

### 2.3. Study procedures

Animal care staff recorded food intake and completed a stool diary, documenting stool frequency, consistency, and characteristics, beginning on study Day 1. Intake was measured twice daily for each animal in each 4-week period. The two daily measurements were summed to provide a total daily intake estimate for each animal. The daily totals were then averaged over each 4-week period to provide an average daily intake for each animal in each period.

Body weight, fecal scores, fecal composition, SCFAs, and fecal and serum metabolomics data were collected at four time points: (1) at the end of the 4-week wash-in period prior to Period 1 (Day 29); (2) at the end of Period 1 (Day 56); (3) at the end of the 4-week washout period between periods 1 and 2 (Day 84); and (4) at the end of Period 2 (Day 122). The consistency and characteristics of feces were assessed using a 5-point scale, from 1, where the dog's stool was liquid in form, to 5, in which the dog's stool was solid and >80% firm. To ensure the welfare of the dogs, any dog who consumed <60% of their maintenance energy requirements was removed from the study.

Whole feces were collected, homogenized by planetary centrifugal mixer until visually uniform, snap-frozen in liquid nitrogen, and stored until processing at −80°C. Proximate analyses and analyses of pH, vitamins, amino acids, and SCFAs were conducted using certified official compendial methods in commercial laboratories accredited by the International Organization of Standards. Moisture of fecal samples was assessed by spreading feces in an aluminum pan and allowing it to dry for ~3 h . Ash values were evaluated by weighing a portion of the fecal sample in a small ceramic crucible and heating the sample to 600°C for ~2 h.

Serum chemistry and complete blood count profiles were obtained from fasted dogs at the end of each washout period (Days 29 and 84) and the end of Period 1 and 2 (Days 56 and 122) to monitor the health and response of dogs throughout the study.

### 2.4. Metabolomic profiling

Fecal and serum metabolomics were performed by Metabolon, Inc (Morrisville, NC), as previously described (35). Briefly, samples were subjected to a methanol-based extraction and divided among four methods for analysis on exact-mass Q-Exactive mass spectrometers (MS): “early” and “late” reverse phase (RP)/ultra-high performance liquid chromatography-tandem-MS (UPLC-MS/MS) in positive ion mode, RP/UPLC-MS/MS in negative ion mode, and polar hydrophilic interaction chromatography/UPLC-MS/MS in negative ion mode. Metabolites were identified and elution peaks were integrated using provider-developed software that matched features to an in-house library of authentic and *in silico* standards, followed by automatic and manual quality control. Compounds with missing values (i.e., those measured in some but not all animals) were imputed with the observed minimum for that biochemical. Metabolites that lacked authentic standards, but still demonstrated high confidence in chemical identification are marked with an asterisk (^*^) in the Supplementary material. Metabolites with identical masses (isobar) that could not be resolved by chromatography were named accordingly (e.g., mannitol/sorbitol).

### 2.5. Microbiome evaluation

Total DNA was extracted from frozen fecal samples using the PowerFecal DNA isolation kit (MOBIO, Carlsbad, CA), following the manufacturer's instructions. A sonication step was performed before vortexing the bead tubes with fecal samples horizontally for 15 minutes, as described by Jackson and Jewell (17). PCR amplification was then conducted using the primer pairs 341F and 806R spanning the V3–V4 hypervariable regions of the 16S RNA gene along with Illumina adapters. Amplicon sequencing was performed according to the Illumina 16s metagenomic sequencing library preparation protocol (15044223 Rev. A). Resulting sequences were de-multiplexed based on the dual index sequences, using the Miseq built-in metagenomics workflow to generate FASTQ files. FASTQ files were processed using Mothur (version 1.39.5) with standard parameters (36, 37). All retained sequences were aligned to Green Genes Database and classified using the naive Bayesian classifier within Mothur with a minimum confidence of 80% for each assignment. Operational taxonomic units were identified based on taxonomic hierarchy and further processed using the Phylogenetic Investigation of Communities by Reconstruction of Unobserved States (PICRUSt) protocol (38) to correct 16s gene copy numbers followed by predicting functional attributes using the Kyoto Encyclopedia of Genes and Genomes (KEGG) database.

The interaction of fecal metabolites and hindgut microbial enzyme functions was assessed by co-mapping those metabolites having compound identifiers and PICRUSt-derived functions having KEGG orthology (KO) identifiers related to tryptophan metabolism, as previously described (16). The resulting relative abundances were projected onto a single space (ko00380; tryptophan metabolism) (36, 37, 39).

### 2.6. Statistical analysis

#### 2.6.1. Evaluation of the effect of health status

Preliminary analyses of the microbiome, metabolome, and SCFA data were performed that included disease group (healthy vs. CGE dogs) and a disease group x diet interaction term as fixed effects in the models. In comparing the fecal metabolites for TF1 and TF2, the disease group main effect was statistically significant for one out of the 376 metabolites analyzed, while disease group x diet interaction was statistically significant for 26 metabolites (rejection rate of 6.9%, which is only slightly higher than the Type I error rate), distributed randomly among eight different metabolomic categories. For the serum metabolites, the disease group main effect was statistically significant for two out of 201 metabolites (1%) analyzed, found in two different metabolomic categories, while a statistically significant disease group × diet interaction was observed for 16 metabolites (8.0%), occurring among eight different metabolomic categories. In comparing TF1 and CF, the disease group main effect was not significant for any fecal metabolites and was statistically significant for only one serum metabolite. The disease group x diet interaction term was statistically significant for 12 fecal metabolites (3.2%), distributed among eight different metabolomic categories, and four serum metabolites (2.0%), which were found in four different categories. Among fecal SCFA, no disease main effects were observed comparing either diet, while a significant disease x diet interaction was observed for propionic acid in comparing TF1 and CF. The lack of a clear and consistent disease effect from these data streams prompted the removal of healthy-CGE status from the final analysis.

#### 2.6.2. Microbiome analysis

Fecal microbiome count data were analyzed at the phylum, family, and genus levels, whereas the alpha-diversity indices and PICRUSt-predicted KO functional data were analyzed at the genus level only. The alpha-diversity indices were analyzed using the Wilcoxon signed-rank test. The 16S copy number-corrected operational taxonomic unit counts and KO functional data were first filtered by prevalence, needing to pass 80% prevalence in at least one of the diet-health experimental groups. KO functions were secondly filtered by an internally curated list of pathways to be considered for further statistical analysis. The counts of individual operational taxonomic units and predicted KO functions were analyzed by negative binomial mixed models (40) with fixed diet and health group effects and random animal effect. Permutational multivariate analysis of variance (PERMANOVA) based on Manhattan distance was used to compare microbial relative abundance compositions and pathway functional compositions between diets (41). *P*-values were false discovery rate-adjusted according to the Benjamini and Hochberg procedure (42). All the statistical microbiome analyses were carried out in R-3.3.3 (43).

#### 2.6.3. Metabolomics analysis

Fecal and serum metabolites were categorized into 39 and 20 functional groups, respectively. Two separate analyses were conducted: (1) comparison of TF1 (*n* = 39) vs. TF2 (*n* = 39) utilizing the crossover design, and (2) comparison of TF1 vs. CF. CF samples (*n* = 78) were treated as repeated measures after combining wash-in and washout measurements. Next, the results from the crossover analysis were reviewed for a significant sequence effect, as an indication of a possible carryover effect. A significant sequence effect was detected in 11 of 373 (2.9%) fecal metabolites examined, and in three of 201 serum metabolites examined (1.5%), both of which were below the 5% null hypothesis rejection rate expected by chance alone, indicating that by the end of each 4-week feeding period there was little or no carryover effect from the previous feeding period present in the fecal metabolites.

For TF1 vs. TF2, the data were analyzed using a linear mixed-model with sequence, treatment, and period as fixed-effects, and animal nested within sequence as a random-effect. For TF1 vs. CF, the data were analyzed using a linear mixed-model with treatment (TF1 and CF) as a fixed-effect and animal and animal × diet as random effects. This analysis treats the measurements taken during the wash-in period and washout period as repeated measurements and fits a compound symmetry covariance structure to the data to account for correlations between the repeated measurements. The Kenwood-Roger adjustment was used to adjust the denominator degrees-of-freedom of the *F*-test for the test of fixed-effects. The NOBOUND option was used in situations where the animal (sequence) [TF1 vs. TF2], the animal or animal × diet [TF1 vs. CF] variance component was negative to allow for negative estimated values so that the degrees-of-freedom associated with this variance component were not pooled with the residual error degrees-of-freedom. Treatment least-squares means and mean differences were estimated using this linear model.

Functional metabolomic groups were analyzed using a multivariate analysis of variance (MANOVA) option in PROC GLM in SAS^®^. The same linear mixed-models described above were used except that PROC GLM does not allow for the use of the Kenward-Roger option. With the crossover analysis of TF1 vs. TF2, the sequence fixed-effect was tested using animal (sequence) as the error term and the treatment and period fixed-effects were tested using the residual error term. With the comparison of TF1 vs. the CF, the residual error term was used to test for a significant diet effect. The Wilk's lambda test statistic was used to identify significant treatment differences. The analysis was performed on scaled variables; therefore, the data were not log-transformed prior to analysis.

Principal component (PC) analysis was performed using PROC PRINCOMP using the correlation matrix to standardize the variables. Scores for the top 9 PCs ranked by percentage of variance were analyzed individually using the linear mixed models described above. Separate analyses were performed for fecal and serum metabolites. All analyses were performed using SAS^®^ PROC MIXED, version 9.4.

## 3. Results

### 3.1. Demographics, study disposition, stool quality, and stool composition

A total of 42 dogs were enrolled and 3 were removed from the study during the initial wash-in period due to megaesophagus on Day 1, pica on Day 15, and intestinal hemorrhage on Day 18. Because these dogs were removed from the study prior to the initial crossover treatment period, data are available on the 39 dogs (healthy, *n* = 21; CGE, *n* = 18) that participated in a randomized crossover design study.

Although study dogs consumed a similar amount of each food, in terms of both grams and calories (Table 3), consumption of TF1 resulted in ~0.3 kg loss of body weight (~2.5%) compared to either CF or TF2 (*P* < 0.0001). Stool scores were evaluated as proxy measurements for gastrointestinal health. Both TF1 (*P* = 0.0002) and TF2 (*P* = 0.01) significantly improved fecal scores relative to CF, while TF1 and TF2 had similar stool scores.

**Table 3:** Mean fecal scores, body weight, and food intake measurements for canines in study.

**Measurement**	**CF ±SE**	**TF1 ±SE**	**TF2 ±SE**	**TF1–CF ±SE**	**TF1–TF2 ±SE**	**TF2–CF ±SE**
*N*	78	39	39			
Fecal score	4.3 ± 0.1	4.8 ± 0.1	4.6 ± 0.1	0.5 ± 0.1^**^	0.1 ± 0.1	0.4 ± 0.1^*^
Body weight (kg)	11.9 ± 0.4	11.7 ± 0.4	11.9 ± 0.4	−0.27 ± 0.05^***^	−0.27 ± 0.06^***^	−0.01 ± 0.05
Intake (g)	212 ± 9	211 ± 9	220 ± 9	0 ± 4	−8 ± 6	8 ± 4
Intake (Kcal)	722 ± 29	706 ± 32	732 ± 32	−15 ± 14	−26 ± 22	11 ± 15

^*^P < 0.05; ^**^P < 0.001; ^***^P < 0.0001. CF, control food; SE, standard error; TF1, test food 1; TF2, test food 2.

Because we did not match a control for TF2 lacking the pre-biotic fiber bundle, we directly compared CF and TF2 only for macroscopic measurements and not for assessments of stool composition, metabolomics, or the microbiome. An analysis comparing the impact of CF, TF1, and TF2 in healthy dogs compared to those with managed CGE revealed no differences in stool quality and very few significant differences in the microbiome, metabolome, or SCFA response to foods by health status (see Materials and methods). Consequently, results for the subgroups of dogs with managed CGE and healthy dogs are not presented in this manuscript. Additionally, a statistical analysis of the metabolomics data from feeding the CF during the wash-in period and washout period indicated the absence of a carryover effect, allowing the wash-in and washout feedings to be combined in the CF analysis.

**Figure 1:**
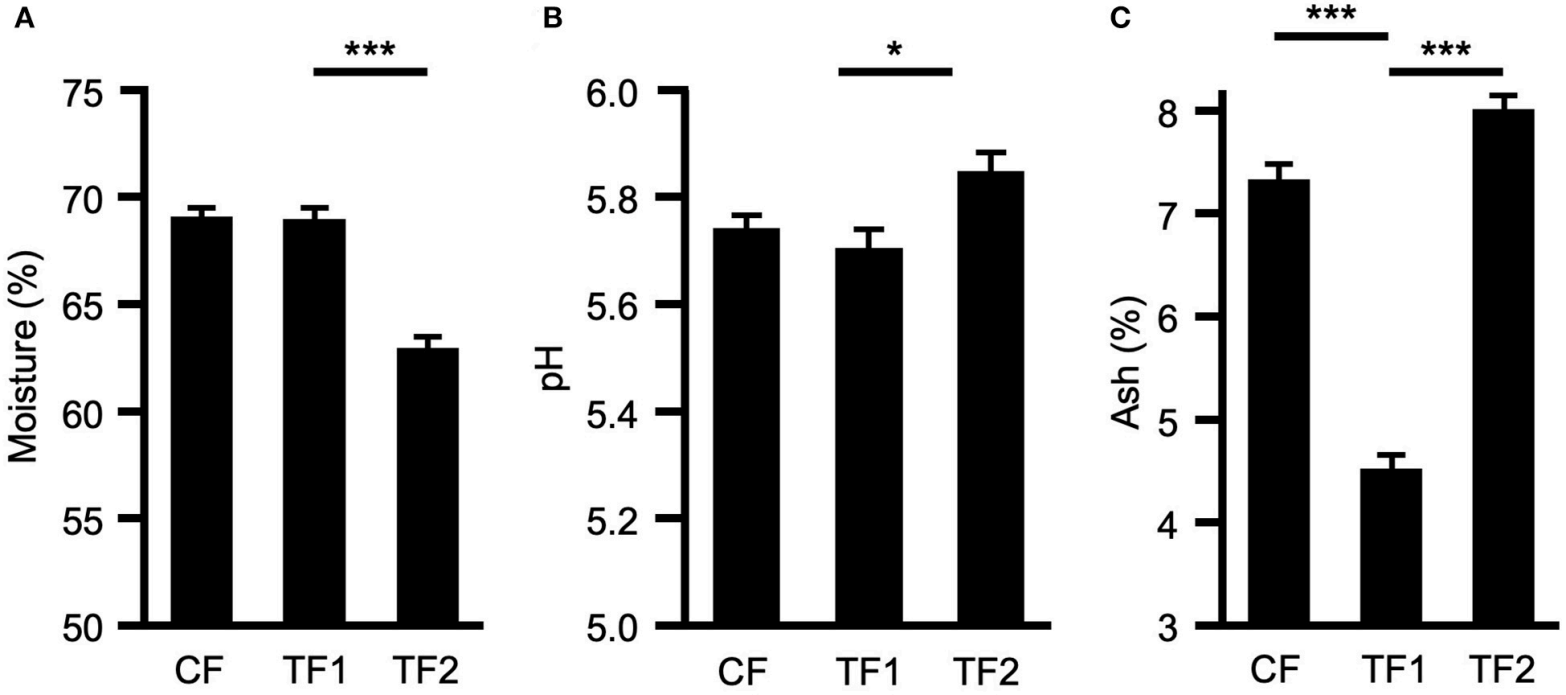
Macroscopic fecal composition comparisons. Fecal measurements for **(A)** percent moisture, **(B)** pH levels, and **(C)** percent ash for dogs consuming control food (CF), test food 1 (TF1), and test food 2 (TF2). Significant differences among CF vs. TF1 and TF1 vs. TF2 are indicated (*P < 0.05, ***P < 0.0001).

TF1 had no effect on either moisture content (Figure 1A) or fecal pH (Figure 1B), compared to CF; however, dogs consuming TF2 defecated stools of significantly lower moisture content (*P* < 0.0001) and significantly higher pH (*P* = 0.001) compared to TF1. TF1 consumption significantly reduced total fecal ash compared to both CF and TF2 (*P* < 0.0001, Figure 1C). Further inspection of the mineral contributions to the ash indicated that, compared to CF, TF1 significantly reduced fecal calcium, copper, iron, magnesium, manganese, phosphorus, and zinc, while significantly increasing fecal sodium levels (Supplementary material 2). Compared to TF2, however, TF1 had significantly higher levels of calcium, copper, potassium, magnesium, and zinc, while manganese and phosphorus levels were significantly lower, and iron and sodium levels were not statistically different.

### 3.2. Fecal microbiome characterization and high-level metabolomics analysis

**Figure 2:**
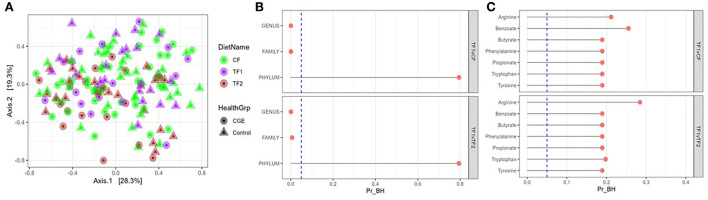
Fecal microbiome analysis. **(A)** Principal coordinate analyses of the fecal microbiome at genus level of dogs consuming different foods used in this study. **(B)** PERMANOVA analyses showing false discovery rate-corrected bacterial taxa (genus, family, and phylum level) and **(C)** KEGG pathways in feces from dogs that consumed the study foods TF1 vs. CF and TF1 vs. TF2. Vertical dotted line represents *P* = 0.05. KEGG, Kyoto Encyclopedia of Genes and Genomes; PERMANOVA, permutational multivariate analysis of variance; Pr_BH, *p*-value corrected by the Benjamini–Hochberg method; CF, control food; TF1, test food; TF2, test food 2.

For fecal microbiome abundance data, five phyla, 41 families and 80 genera passed the prevalence filter and were considered for statistical analyses. The results of principal coordinate analyses based on beta diversity measures using Manhattan distance matrix are shown in Figure 2A; no visible clustering was observed for food type (CF, TF1, and TF2) or health status (healthy and CGE). By contrast, PERMANOVA analysis revealed significant compositional differences by treatment between both TF1 and CF (*P* = 0.02) and TF1 and TF2 (*P* = 0.005), at the family and genus levels, but not at the phylum level (Figure 2B).

Analysis of individual phyla indicated that Fusobacteria significantly increased (*P* = 0.01) in dogs fed TF1 compared with CF and significantly decreased (*P* < 0.0001) in dogs fed TF1 compared with TF2 (Supplementary material 3). Similarly, analyses of individual families and individual genera showed that the relative abundances of family Pseudomonadaceae, and genus *Pseudomonas*, which belong to the phylum Proteobacteria, significantly decreased in the dogs fed TF1 compared to both CF and TF2, as well as family Clostridiaceae and genera *Clostridium* and 02d06, which belong to phylum Firmicutes. Additional Firmicutes family Ruminococcaceae and genus *Faecalibacterium* were significantly increased in dogs fed TF1 compared with CF and TF2. Families such as Bacteroidaceae, Turicibacteraceae, Peptostreptococcaceae, and Fusobacteriaceae, as well as genera *Bacteroides, Turicibacter*, and *Megamonas* were significantly increased in dogs fed TF1 compared with CF. The Proteobacteria family Helicobacteraceae and genus *Helicobacter*, as well as the Firmicutes genera *Blautia* and *Dialister* were significantly increased in dogs fed TF1 compared with TF2.

All calculated bacterial -diversity indexes at the genus level, including the Shannon diversity index and the Simpson index, showed no significant differences among either CF and TF1 or TF1 and TF2. Richness analysis indicated that the number of species within a given sample were significantly reduced in TF1 vs. either CF (*P* = 0.005) or TF2 (*P* = 0.004). PERMANOVA analyses of microbial KO functional compositions showed no significant differences between the food comparisons TF1 vs. CF and TF1 vs. TF2 for all curated pathways (Figure 2C).

We performed PC analysis on both the fecal and serum metabolomics datasets for all collected samples. Eigenvalues and proportion of variation explained by the top 9 PCs for the fecal and serum datasets are presented in Supplementary material 4. Overall, PC1 accounted for 12.4 and 10.6% of the variation in fecal and serum metabolites, respectively, while PC2 accounted for 9.9 and 7.5% of the total fecal and serum metabolites, respectively. Approximately half of the metabolites with relatively high loadings contributing to PC1 were from the amino acid superpathway, while about two thirds of those contributing to PC2 were from the lipid superpathway.

**Figure 3:**
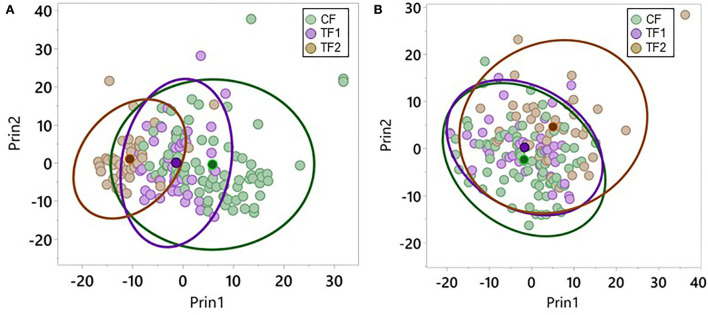
Principal component analysis for metabolomics data. Individual dogs consuming CF (green), TF1 (violet), and TF2 (brown) were plotted according to their aggregate PC1 and PC2 scores for **(A)** feces and **(B)** serum. Diet group centroids and 95% confidence intervals are indicated by darker dots and ovals, respectively.

Plotting the top 2 PCs in the fecal dataset by the diets each individual animal consumed revealed significant (*P* < 0.0001) separation in PC1 when comparing CF and TF1 and when comparing TF1 and TF2; however, such differences were not observed in PC2 (Figure 3A). Similar analyses performed using the serum dataset indicated significant (*P* < 0.0001) separation in both PC1 and PC2 between TF1 and TF2, but only the difference between CF and TF1 in PC2 reached statistical significance (*P* < 0.0001; Figure 3B).

### 3.3. Impact of fiber inclusion on microbial metabolism

#### 3.3.1. Saccharolytic metabolism

**Figure 4:**
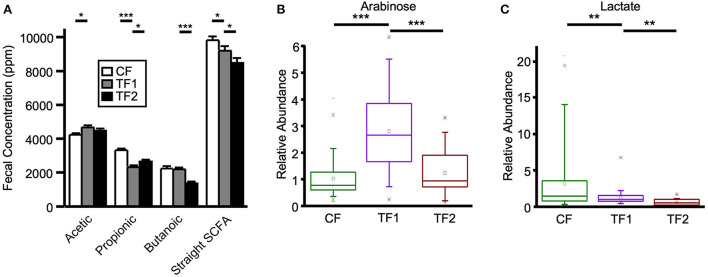
Fecal markers of saccharolytic microbial metabolism. **(A)** Straight short chain fatty acid measurements for dogs consuming CF (white), TF1 (gray), and TF2 (black). Relative abundance levels for **(B)** arabinose and **(C)** lactate in healthy dogs consuming CF (green), TF1 (violet), and TF2 (brown). Significant differences among CF vs. TF1 and TF1 vs. TF2 are indicated (*P < 0.05, **P < 0.001, ***P < 0.0001).

Fecal straight SCFA quantitative measurements (Figure 4A), as well as monosaccharide metabolomic relative abundances, were analyzed as surrogates for microbial saccharolytic processes. TF1-fed dogs showed significantly increased fecal acetic acid levels (*P* = 0.001) while significantly reducing propionic acid (*P* < 0.0001), and an overall decrease in total straight SCFA levels compared to CF-fed dogs (*P* = 0.03). Compared to dogs in the TF2 group, dogs in the TF1 group showed significantly reduced fecal propionic acid levels (*P* = 0.03) but significantly higher butanoic acid levels (*P* < 0.0001), ultimately resulting in higher total straight SCFA levels in the TF1 group vs. the TF2 group (*P* = 0.04).

TF1 ingestion significantly affected both fecal (Table 4) and serum (Table 5) carbohydrates (*P* < 0.0001 by MANOVA) compared to CF, yet the directionality was metabolite dependent. In feces (Supplementary material 5), consumption of TF1 resulted in significantly elevated arabinose (Figure 4B), galacturonate, and ribulose/xylulose [isobar] levels compared to CF (all *P* < 0.0001). Fecal levels of erythritol (*P* = 0.004), lactate (*P* = 0.009; Figure 4C), mannitol/sorbitol [isobar] (*P* = 0.03), ribose (*P* = 0.0001), and xylose (*P* < 0.0001) were all decreased with TF1 vs. CF. Serum assessments of these metabolites (Supplementary material 6) revealed significant reductions in circulating erythritol (*P* = 0.0008), lactate (*P* < 0.0001), and ribose (*P* = 0.001) in TF1-fed dogs vs. CF. Glucuronate (*P* = 0.02) and xylose (*P* = 0.02) were significantly elevated with TF1 consumption in serum vs. CF, while mannitol/sorbitol was unchanged. Reductions in serum erythronate (*P* = 0.004) and pyruvate (*P* < 0.0001) were observed, yet the addition of the fiber bundle in TF1 had no appreciable effect on these metabolites in feces.

**Table 4:** Overall fecal metabolomics data^a^.

**Metabolite classification**		**TF1 vs. CF**	**TF1 vs. TF2**
		**MANOVA** ***p*****-value**	**MANOVA** ***p*****-value**
Sugars		<0.0001	<0.0001
Putrefactive metabolism	Amino acids	<0.0001	<0.0001
	Dipeptides	<0.0001	<0.0001
	Polyamines	<0.0001	<0.0001
Collagen metabolism		0.0001	<0.0001
Tryptophan metabolism	Indole pathway	<0.0001	<0.0001
	Kynurenine pathway	<0.0001	<0.0001
	Serotonin pathway	0.0003	<0.0001
Plant-based compounds	Alkaloids	<0.0001	<0.0001
	Benzoate metabolism	<0.0001	<0.0001
	Phenolics	<0.0001	0.0003
	Post-biotics	<0.0001	<0.0001
	Terpenoids	<0.0001	<0.0001
Endocannabinoids		<0.0001	<0.0001
Free fatty acids	Polyunsaturated n3	<0.0001	<0.0001
	Polyunsaturated n6	0.0184	<0.0001
	Saturated & monounsaturated	<0.0001	<0.0001
Acylglycerols	Monoacylglycerols	<0.0001	<0.0001
	Diacylglycerols	<0.0001	<0.0001
Linolenate metabolism		<0.0001	<0.0001
Sphingolipids	All	<0.0001	0.0006
	Ceramides	<0.0001	<0.0001
	Dihydroceramides	0.0046	0.0004
	Dihydrosphingomyelins	0.0686	0.0016
	Hexosylceramides (HCER)	<0.0001	<0.0001
	Lactosylceramides (LCER)	<0.0001	<0.0001
	Sphingolipid synthesis	0.0019	0.3686
	Sphingosines	0.0144	<0.0001
	Sphingomyelins (SM)	0.0003	0.0026
Phospholipids	Lysophospholipids	<0.0001	<0.0001
	Phosphatidylcholines (PC)	<0.0001	<0.0001
	Phosphatidylethanolamines (PE)	<0.0001	0.0002
	Phospholipid Metabolism	<0.0001	<0.0001
Vitamins and cofactors	Hemoglobin Metabolism	<0.0001	0.1841
	NAD Metabolism	<0.0001	<0.0001
	Tocopherol Metabolism	<0.0001	<0.0001
Bile acids	Primary	<0.0001	0.0149
	Secondary	0.0002	0.0069
Redox active couples	Oxidized	0.0042	0.0052
	Reduced	0.0085	<0.0001

^a^For each test, blue and red indicate metabolites within the pathway significantly increased or decreased in the dogs consuming TF1 relative to the comparator, respectively, as determined by summation of the numerical values for individual metabolites within the overall class. Yellow indicates the pathway was significantly altered but overall directionality was inconclusive. Full numerical data are delineated in Supplementary material 5. CF, control food; MANOVA, multivariate analysis of variance; TF1, test food 1; TF2, test food 2.

**Table 5:** Overall serum metabolomics data^a^.

**Metabolite classification**		**TF1 vs. CF**	**TF1 vs. TF2**
		**MANOVA** ***p*****-value**	**MANOVA** ***p*****-value**
Sugars		<0.0001	<0.0001
Amino acids		<0.0001	<0.0001
Tryptophan indole pathway		0.0004	<0.0001
Free fatty acids	Eicosanoids	0.4516	0.8588
	Polyunsaturated n3	<0.0001	<0.0001
	Polyunsaturated n6	0.0009	<0.0001
	Saturated & monounsaturated	0.0012	<0.0001
Sphingolipids	All	<0.0001	<0.0001
	Ceramides	<0.0001	<0.0001
	Dihydroceramides	0.1211	0.0096
	Dihydrosphingomyelins	0.0006	<0.0001
	Hexosylceramides (HCER)	0.0003	<0.0001
	Lactosylceramides (LCER)	0.2302	<0.0001
	Sphingosines	0.0687	0.2188
	Sphingomyelins (SM)	0.0001	<0.0001
Phospholipids	Lysophospholipids	<0.0001	<0.0001
	Phosphatidylcholines (PC)	<0.0001	<0.0001
	Phosphatidylethanolamines (PE)	0.0001	<0.0001
	Phosphatidylinositol (PI)	<0.0001	<0.0001
Tocopherol metabolism		<0.0001	<0.0001
Collagen metabolism		<0.0001	<0.0001

^a^For each test, blue and red indicate metabolites within the pathway significantly increased or decreased in the dogs consuming TF1 relative to the comparator, respectively, as determined by summation of the numerical values for individual metabolites within the overall class. Yellow filled cells indicate the pathway was significantly altered but overall directionality was inconclusive. Full numerical data are delineated in Supplementary material 7. CF, control food; MANOVA, multivariate analysis of variance; TF1, test food 1; TF2, test food 2.

Compared to TF2, TF1 consumption resulted in significantly higher levels of both fecal and serum carbohydrate metabolites (*P* < 0.0001 by MANOVA). Both fecal and serum analyses revealed that consumption of TF1 increased erythronate and lactate, while significantly reducing levels of mannose, compared to TF2. Other fecal monosaccharides higher in TF1-fed dogs included arabinose, all galactose-related compounds, glucose, glycerate, maltose, mannitol/sorbitol [isobar], pyruvate, and ribulose/xylulose [isobar]. Xylose was the only monosaccharide lower in dogs consuming TF1 compared to TF2.

#### 3.3.2. Putrefactive metabolism

**Figure 5:**
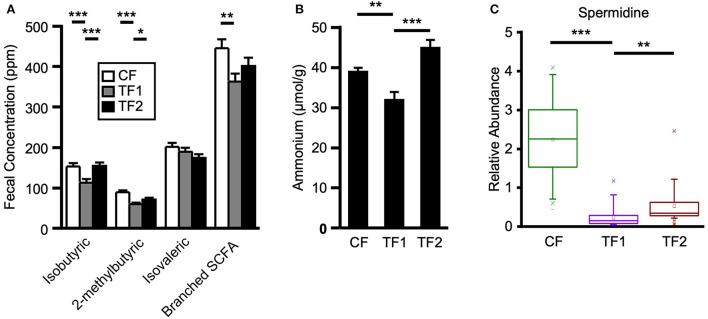
Markers of proteolytic microbial metabolism. **(A)** Branched short chain fatty acid measurements for dogs consuming CF (white), TF1 (gray), and TF2 (black). **(B)** Ammonium levels and **(C)** spermidine relative abundance levels for adult dogs consuming CF (green), TF1 (violet), and TF2 (brown). Significant differences among CF vs. TF1 and TF1 vs. TF2 are indicated (*P < 0.05, **P < 0.001, ***P < 0.0001).

To evaluate dietary contributions to putrefactive microbial metabolism, quantitative measurements of fecal branched SCFAs and ammonium, as well as relative abundances of amino acids, dipeptides, and polyamines were considered. TF1 consumption significantly reduced fecal isobutyric (*P* < 0.0001) and 2-methylbutyric (*P* < 0.0001) acids from CF levels, and both branched SCFAs were also significantly lower in the stool of TF1-fed dogs compared to TF2-fed dogs (Figure 5A). Total fecal branched SCFAs were significantly reduced in dogs consuming TF1 compared to CF (*P* = 0.0008). Fecal total branched SCFAs were lower in TF1-fed dogs relative to TF2-fed dogs, although this difference was not statistically significant (*P* = 0.08). Fecal ammonium concentrations (Figure 5B) in dogs fed the fiber bundle in TF1 were lower than those in CF-fed dogs (*P* = 0.0001) and in TF2-fed dogs (*P* < 0.0001).

Compared to CF, TF1 was associated with significant reductions in both fecal (Table 4) and serum (Table 5) levels of amino acids, as well as fecal polyamines (all *P* < 0.0001), as indicated by MANOVA. The amino acids alanine, cysteine, methionine, and proline were reduced in both feces (Supplementary material 5) and serum (Supplementary material 6) in dogs fed TF1 vs. CF, while isoleucine, lysine, threonine, and valine were only reduced in feces. Significant differences in fecal and serum amino acids were also observed between TF1 and TF2 (Table 4; all *P* < 0.0001). Among the 21 fecal amino acids evaluated, 18 were significantly elevated among dogs fed TF1 compared to TF2, while levels of aspartate, proline, and taurine were similar. Yet when comparing circulating amino acids in dogs fed TF1 vs. TF2, 9 were higher with TF1, including aspartate (*P* = 0.0006), and five were lower, including taurine (*P* = 0.0008).

Among the 18 fecal dipeptides, seven were higher and six were lower in stools of dogs fed TF1 vs. CF. When fecal dipeptide levels were compared between TF1 and TF2, the levels of 14 were significantly higher in TF1-fed dogs.

Almost all measured fecal polyamines and associated metabolites were significantly reduced in dogs fed TF1 vs. CF, including cadaverine, putrescine, and spermidine (Figure 5C; all *P* < 0.0001). Fiber inclusion in TF1 also significantly reduced the N-acetylated metabolites of these primary polyamines, including N1, N12-diacetylspermine vs. CF, while spermine was not observed in feces. Exceptions included ornithine, the precursor to putrescine, and the primary polyamine agmatine, which were both unchanged. Differences in fecal polyamines were inconsistent among TF1 and TF2; the metabolic intermediates 4-acetamidobutanoate, 5-methylthioadenosine, and ornithine were significantly higher in TF1-fed dogs, as were N-acetyl-cadaverine and N-acetylputrescine, while carboxyethyl-GABA and [N(1) + N(8)]-acetylspermidine levels were significantly lower. The primary polyamine spermidine was significantly lower in the stools of TF1-fed dogs vs. TF2-fed dogs (*P* = 0.0001), yet this decrease (0.30) was less pronounced than the difference between CF- and TF1-fed dogs (2.00, *P* < 0.0001; Figure 5C).

#### 3.3.3. Tryptophan metabolism

Metabolism of the essential amino acid tryptophan was implicated as a potential driving factor in TF1 alleviating clinical diarrhea in our previous longitudinal, single-arm intent-to-treat study (16). In this study, TF1 feeding did not appreciably affect fecal tryptophan levels vs. CF, yet it significantly reduced tryptophan-related metabolites in the kynurenine (*P* < 0.0001), serotonin (*P* = 0.0003), and indole (*P* < 0.0001) subpathways in feces (Table 4, Supplementary material 5). Levels of the kynurenine-related metabolites 2-aminophenol (*P* < 0.0001), anthranilate (*P* = 0.0001), N-formylanthranilic acid (*P* = 0.01), picolinate (*P* < 0.0001), and quinolinate (*P* = 0.03) were all significantly reduced in TF1- vs. CF-fed dogs, while kynurenine, kynurenate, and xanthurenate levels were unchanged. Serotonin (*P* = 0.04) and its catabolite 5-hydroxyindoleacetate (*P* = 0.001) were both significantly reduced with TF1 from CF-fed levels. Significantly reduced fecal indoles in TF1-fed dogs vs. CF-fed dogs included 2-oxindole-3-acetate (*P* < 0.0001), indole (*P* = 0.01), and tryptamine (*P* = 0.004), as well as indolelactate (*P* = 0.006) and indolin-2-one (*P* = 0.0001). Levels of the latter two compounds, as well as 3-indoxyl sulfate (*P* = 0.006) and tryptophan (*P* < 0.0001), were among indoles measured in both feces and serum that were also reduced in circulation, while metabolites in the tryptophan indole pathway were significantly reduced (*P* = 0.0004) in dogs consuming TF1 relative to CF (Table 5, Supplementary material 6).

**Figure 6:**
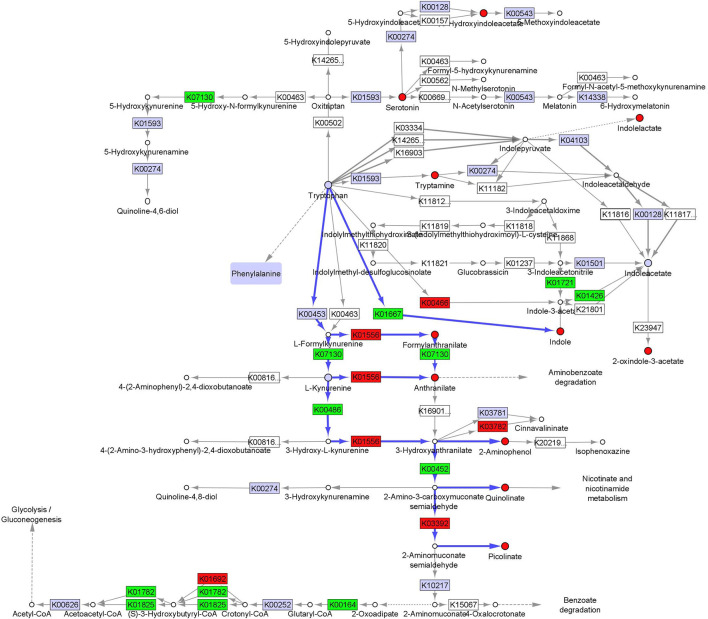
Fecal microbiome and metabolomic pathway analysis of tryptophan. Simplified projection of fecal metabolite and inferred function from microbiome measurements on the KEGG tryptophan pathway (KO00380) (36, 37, 39). Significantly increased and decreased enzymatic functions and metabolites are colored green and red, respectively, while observed but unchanged functions and metabolites are rendered gray. Likely catabolic paths discussed in the text are indicated with blue arrows.

We integrated fecal inferred microbial enzyme functions from PICRUSt and tryptophan metabolites to project relative abundance differences between CF and TF1 consumption onto a simplified tryptophan metabolic pathway map (KO00380, Figure 6, Supplementary material 7). The projection illustrates how TF1 feeding particularly impacted microbial metabolism of tryptophan through kynurenine metabolism to nicotinamides and energy substrates (e.g., 2-oxoglutarate dehydrogenase, K00164, *P* < 0.0001) in the tricarboxylic acid cycle. TF1 consumption resulted in increased kynurenine-3-monooxygenase (K00486, *P* < 0.0001) and 3-hydroxyanthranilate 3,4-dioxygenase (K00452, *P* < 0.0001) catabolism of kynurenine, as well as arylformidase (K07130, *P* = 0.02) catabolism of N-formylanthranilic acid, while activity of downstream enzymes kynureninase (K01556, *P* = 0.01) and aminocarboxymuconate-semialdehyde decarboxylase (K03392, *P* = 0.0001) were downregulated. In the indole pathway, tryptophanase (K01667, *P* < 0.0001) activity was elevated in TF1-fed dogs, as were nitrilase (K01501) and amidase (K01426, *P* = 0.008) functions, while tryptophan 2-monooxygenase (K00466) abundance was downregulated.

Fecal tryptophan levels were elevated and activity in all three of its metabolic subpathways were significantly upregulated in dogs consuming TF1 compared to dogs consuming TF2 (*P* < 0.0001). Notable fecal tryptophan metabolites that were increased in TF1-fed dogs include kynurenate (*P* = 0.02), kynurenine (*P* < 0.0001), serotonin (*P* < 0.0001), 2-oxindole-3-acetate (*P* < 0.0001), indoleacetate (*P* < 0.0001), indoleacetylglutamine (*P* = 0.005), and indolepropionate (*P* = 0.0001). Circulating levels of indoleacetylglutamine and indolepropionate were also significantly higher in dogs consuming TF1 vs. TF2, and serum indoles as a class were significantly different (*P* < 0.0001), despite serum tryptophan levels being statistically similar. Levels of the fecal tryptophan metabolites N-formylanthranilic acid (*P* = 0.0008), quinolinate (*P* = 0.0002), and indolin-2-one (*P* = 0.008) were significantly lower in TF1-fed dogs vs. TF2-fed dogs. Circulating 3-indoxyl sulfate (*P* = 0.001) and indolin-2-one (*P* = 0.004) levels were also significantly reduced in dogs fed TF1, compared to those fed TF2.

### 3.4. Impact of the pre-biotic fiber bundle on the availability of inflammatory mediators and anti-oxidative plant-derived compounds

#### 3.4.1. Plant-derived phenolics, alkaloids, and terpenoids, and corresponding post-biotics

Nutritional plant components measured in feces were delineated as phenolics, alkaloids, and terpenoids (Supplementary material 5). MANOVA indicated that phenolic compounds were significantly enriched (*P* < 0.0001) in the feces of dogs consuming TF1 compared to both CF and TF2 (Table 4). Most of these biochemicals were increased by roughly the same amount in dogs fed TF1 relative to those fed CF or TF2, which likely reflects their inclusion in the fiber bundle. Among the citrus-derived flavone and flavonoid polyphenols enriched in the stool of TF1-fed dogs vs. TF2- and CF-fed dogs were apigenin, chrysoeriol, diosmetin, sinensetin, and eriodictyol, as well as the flavanone glycosides hesperidin, and narirutin. The dihydrochalcone phloretin was also similarly significantly elevated (*P* < 0.0001) in the stool of dogs fed TF1 relative to both CF and TF2. Similarly, the flaxseed-rich lignan secoisolariciresinol diglucoside was also elevated in the stool of TF1-fed dogs (*P* < 0.0001), relative to CF and TF2. Conversely, levels of the soy-sourced isoflavones daidzein and genistein were similar among CF and TF1 fecal samples but were significantly reduced in the stools of TF1-fed dogs compared to those of TF2-fed dogs, while glycitein levels were similar between TF1- and CF-fed dogs and between TF1- and TF2-fed dogs. Additionally, fecal levels of the phenolic acids sinapate, syringic acid, and vanillate were similar in TF1- and CF-fed dogs and significantly elevated in dogs consuming TF1 relative to TF2, indicating these were also likely components of CF.

**Figure 7:**
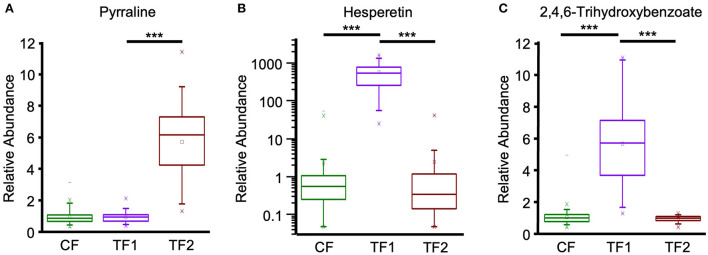
Fecal plant-derived compounds and microbial post-biotics metabolism. Relative abundance levels for **(A)** pyrraline, **(B)** hesperetin, and **(C)** 2,4,6-trihydroxybenzoate in dogs consuming CF (green), TF1 (violet), and TF2 (brown). Significant differences among CF vs. TF1 and TF1 vs. TF2 are indicated (***P < 0.0001).

Multivariate analysis indicated that fecal alkaloid (*P* < 0.0001) and terpenoid (*P* < 0.0001) levels were both significantly different among TF1-fed dogs compared to dogs fed either CF or TF2 (Table 4). Piperidine and stachydrine levels were significantly higher (*P* < 0.0001) with TF1 relative to both CF and TF2, pointing to their enrichment in TF1. Deoxymugineic acid levels were higher in stools of TF1-fed dogs compared to TF2-fed dogs but were similar to those observed in the CF group. Conversely, both TF1- and CF-fed dogs had similar fecal levels of pyrraline; however, TF1-fed dogs had significantly lower levels (*P* < 0.0001) than TF2-fed dogs (Figure 7A). Dogs in the TF1 group also showed significantly reduced fecal levels of DIMBOA (2,4-dihydroxy-7-methoxy-1,4-benzoxazin-3-one), 1-methyl-beta-carboline-3-carboxylic acid, and nicotianamine relative to CF, while fecal levels of DIMBOA and 1-methyl-beta-carboline-3-carboxylic acid in TF-1 fed dogs were significantly higher than those in TF2-fed dogs. TF1 feeding significantly reduced all the fecal steroidal terpenoids (beta-sitosterol, campesterol, ergosterol, lanosterol, and stigmasterol) and the fecal carotene diols relative to CF. By contrast, levels of these compounds were significantly higher in TF1-fed dogs vs. TF2-fed dogs, with the exception of ergosterol, which was significantly lower for TF1, and lanosterol, which showed no significant differences between groups. Fecal limonoids limonin and nomilin were both significantly enriched in TF1-fed dogs relative to either CF-fed or TF2-fed dogs, as was the case for the chlorophyll metabolite pheophorbide A.

We separately conducted a multivariate analysis on fecal post-biotics known to arise from the microbial metabolism of plant biochemicals (Table 4). These post-biotics were significantly enriched (*P* < 0.0001 by MANOVA) in TF1-fed dogs relative to dogs fed either CF or TF2. These included the deglycosylated flavonoids hesperetin (Figure 7B), naringenin, and ponciretin, the deglycosylated lignan secoisolariciresinol, and the phytoestrogen enterodiol (Supplementary material 5). In contrast, levels of the other phytoestrogens, enterolactone, and equol, were similar among dogs consuming the three foods. Diaminopimelate and 2-piperidinone were the only compounds in this class that were lower in TF1-fed dogs than in CF-fed dogs but these levels were similar among dogs consuming TF1 and TF2. Beta-guanidinopropanoate levels were lower in TF1-fed dogs vs. TF2-fed dogs, yet similar in TF1-fed dogs vs. CF-fed dogs.

Fecal benzoate metabolism was significantly different (*P* < 0.0001 by MANOVA) in TF1-fed dogs relative to either CF- or TF2-fed dogs (Table 4). Notably, the flavonoid metabolite 2,4,6-trihydroxybenzoate was significantly enriched (*P* < 0.0001) in TF1-fed dogs by roughly the same amount when compared to either CF- or TF2-fed dogs (Figure 7C). To a lesser extent, levels of the related flavonoid metabolite 3,4-dihydroxybenzoate were also significantly higher in stools of TF1-fed dogs relative to CF-fed dogs (*P* < 0.0001) or TF2-fed dogs (*P* = 0.006). Conversely, 3-(2-hydroxyphenyl) propionate levels were similar among dogs consuming TF1 and TF2, but were significantly lower (*P* = 0.009) among TF1-fed dogs vs. CF-fed dogs. Levels of other benzoate-related catabolites were similar among TF1- and CF-fed dogs but were significantly higher in TF1-fed dogs relative to TF2-fed dogs.

#### 3.4.2. Fatty acid lipid mediators

We considered the fecal N-acyl amino acids, including arachidonoyl ethanolamide (AEA) and its N-acylethanolamide (NAE) congeners as endocannabinoids, molecules with diverse functions in the mammalian gut. As a group, fecal levels of these endocannabinoids were significantly reduced (*P* < 0.0001) in TF-fed dogs relative to either CF- or TF2-fed dogs (Table 4). By univariate analysis, TF1 feeding significantly decreased each of the 14 measured fecal NAEs compared to CF, but only one of the six measured taurine- and serine-containing species (Supplementary material 5). Among TF1 and TF2, 11 NAEs were significantly decreased with TF1 vs. TF2, including AEA (*P* < 0.0001), the primary cannabinoid receptor agonist, which showed the largest magnitude reduction in relative abundance (2.84), as were four out of six taurine- and serine-linked fatty acids.

Polyunsaturated fatty acids (PUFAs) are food-derived precursors to lipid mediators, and here we separated n3 and n6 PUFAs for multivariate analysis. We separately analyzed linolenate-containing lipids since the n3 vs. n6 isomerism was not resolved. Linolenate-containing lipids were significantly elevated (*P* < 0.0001) in stools and circulation among TF1-fed dogs compared with either CF- or TF2-fed dogs. TF1 was associated with significantly increased levels of n3 PUFAs compared with CF or TF2 (both *P* < 0.0001, Table 4). Among the eight measured n3 PUFAs in feces, levels of six were significantly higher among TF1-fed dogs vs. CF-fed dogs and five were significantly higher with TF1 than with TF2 (Supplementary material 5), including docosahexaenoate and eicosapentaenoate. Circulating eicosapentaenoate and linolenate were significantly higher in the TF1 group than in either the CF or the TF2 groups.

TF1 feeding also significantly increased both fecal (*P* = 0.02) and serum (*P* = 0.0009) n6 PUFAs relative to CF. Circulating and fecal levels of hexadecadienoate (16:2n6) and linoleate (18:2n6) were significantly elevated among TF1-fed dogs compared to CF-fed dogs, while arachidonate (20:4n6) was only higher with TF1 in feces. Levels of docosapentaenoate (22:5n6) were elevated in stools of TF1-fed dogs relative to CF-fed dogs, yet levels were reduced in TF1-fed dogs vs. CF-fed dogs in circulation. Fecal n6 PUFAs indicated levels were significantly different among the test foods (*P* < 0.0001 by MANOVA). By univariate analysis, fecal hexadecadienoate (16:2n6) levels were lower in TF1-fed dogs vs. TF2-fed dogs, while levels of all other detected n6 PUFAs were similar in the TF1 group relative to either CF or TF1. Circulating n6 PUFAs were significantly higher in TF1-fed dogs vs. CF-fed dogs (*P* = 0.0009) and lower vs. TF-2 fed dogs by MANOVA (*P* < 0.0001). Four of eight circulating n6 PUFAs were significantly lower in TF1-fed dogs (*P* < 0.0001), including arachidonate (20:4n6) and docosapentaenoate (22:5n6). Levels of circulating eicosanoids were comparable among TF1 vs. either CF or TF2.

#### 3.4.3. Sphingolipids

Ingestion of the pre-biotic fiber bundle profoundly impacted fecal sphingolipids, reducing this class of metabolites in TF1-fed dogs vs. CF-fed dogs (*P* < 0.0001), including significant reductions in the ceramide (*P* ≤ 0.0001), dihydroceramide (*P* = 0.005), hexosylceramide (*P* < 0.0001), lactosylceramide (LCER, *P* < 0.0001), sphingosines (*P* = 0.01), and sphingolipid synthesis metabolite (*P* = 0.002) subclasses, as indicated by MANOVA (Table 4). By univariate analysis, 15 of these 18 fecal metabolites showed significant reductions with TF1 vs. CF (Supplementary material 5). Fecal sphingomyelins (SM) as a class were significantly altered (*P* = 0.0003) with the addition of fiber in TF1 vs. CF, yet an increase in tricosanoyl sphingomyelin (d18:1/23:0)^*^ levels was the only difference to reach significance by univariate analysis (*P* < 0.05) compared to CF. Dogs consuming TF1 showed significantly reduced circulating levels of SM, dihydrosphingomyelins, and HCERs compared to CF, as well as significantly altered ceramides and sphingolipids levels overall with inconclusive directionality (Table 5). Circulating LCER levels were similar with TF1 and CF.

While TF1 consumption resulted in an almost universal reduction in fecal sphingolipids compared to CF, multivariate analysis indicated fecal sphingolipid levels were significantly higher in the stool of TF1-fed dogs compared to TF2-fed dogs overall (*P* = 0.0006) and in most subcategories, including SM and dihydrosphingomyelin (Table 5). In contrast to the results in stool, circulating sphingolipids were significantly lower in TF1-fed dogs compared to TF2-fed dogs (*P* < 0.0001 by MANOVA). Such differences were evident in all sphingolipid subcategories except sphingosines, for which sphingosine 1-phosphate levels were unchanged (Supplementary material 6). In fact, univariate analysis revealed that 18 out of 23 detected circulating sphingolipids were significantly lower in the TF1 group compared to the TF2 group. Levels of serum SM were significantly different among dogs consuming TF1 and TF2 (*P* < 0.0001), yet the directionality was metabolite dependent. Of the 27 detected circulating SMs, 13 were lower and 4 were higher among TF1-fed dogs vs. TF2, respectively.

### 3.5. Phospholipids associated with the gut mucosal layer

Multivariate analysis (Table 4) indicated that TF1 feeding significantly decreased fecal phosphatidylcholines (PCs, *P* < 0.0001) and fecal phosphatidylethanolamines (PEs, *P* < 0.0001) compared to CF, and significantly altered other phospholipid metabolites (*P* < 0.0001). Eight of the 11 fecal PCs showed significant reductions with TF1 vs. CF, and all six fecal PEs were also significantly lower when the fiber bundle was included (Supplementary material 5). On the other hand, TF1 consumption significantly increased levels of fecal lysophospholipids (*P* < 0.0001), with 6/13 individual lysophospholipids higher in T1-fed dogs compared to CF-fed dogs. TF1 was associated with significantly increased levels of circulating PCs (*P* < 0.0001), PEs (*P* = 0.0001), and phosphatidylinositols (PIs, *P* < 0.0001), and significantly decreased serum lysophospholipid levels (*P* < 0.0001) compared to CF. Among the 19 circulating PCs, levels of 10 were lower and four were higher in the TF1 group than in the CF group. Notably, nine of the serum PCs that were reduced in TF1-fed dogs were also reduced in feces compared to CF (Supplementary material 6).

Levels of fecal phospholipids were also largely significantly lower with TF1 feeding vs. TF2. TF1 consumption led to significantly lower levels of fecal PCs (*P* < 0.0001) and PEs (*P* = 0.0002) by multivariate analysis, whereas univariate analysis indicated that eight of 11 individual PCs and four of six PEs were significantly reduced with TF1 vs. CF. Circulating PCs and PEs were also significantly different between TF1 and TF2 on multivariate analysis. Of the 19 measured serum PCs, seven were higher and 10 were lower in TF1-fed dogs compared to TF2-fed dogs. Intriguingly, 12 of these significantly altered PCs had the same directionality in comparing TF1 and TF2 as when comparing CF and TF1. A similar effect was observed for PEs, whereby three of the five significantly altered lipids showed the same directionality when TF1-fed dogs were compared to either TF2- or CF-fed groups. Multivariate analysis indicated significantly lower levels of circulating PIs and lysophospholipids among TF1-fed dogs compared to TF2-fed dogs. By univariate analysis, consumption of TF1 resulted in significant reductions in five of six PIs and 11 of 22 lysophospholipids, compared to consumption of TF2, while two additional lysophospholipids were significantly elevated in the TF1 group vs. the TF2 group.

## 4. Discussion

### 4.1. Distinct macroscopic, microbiome, and metabolomic signatures associated with dietary fiber

A veterinary program of managed care for dogs with CGE typically involves nutritional interventions intended to relieve symptoms and relieve colonic dysbiosis. We surmised that the efficacy of therapeutic foods for CGE could be driven by the specific effects of the novel pre-biotic fiber bundle on the gastrointestinal microbiome of the adult dogs consuming the food. This crossover study was designed to test this hypothesis by evaluating the clinical, metabolic, and metabolomic effects of a food containing this novel pre-biotic fiber bundle, a commercially available therapeutic food with traditional fibers, and a control food in healthy adult dogs and dogs with well-managed CGE.

Stool characteristics indicated that TF1 and TF2 improved GI function of the dogs in the study compared to CF, which lacked the novel pre-biotic fiber bundle. Both TF1 and TF2 significantly increased fecal scores compared to CF, which is consistent with the designated indication of these therapeutic foods. The pre-biotic fiber bundle technology comprises both soluble and insoluble fibers, specifically chosen and proportioned for their unique properties, including prebiotic, water holding, stool bulking, and antioxidant characteristics (16, 30). Inclusion of the pre-biotic fiber bundle had no effect on moisture content relative to CF, yet it still improved stool scores, which is reflective of the selected fiber properties. Furthermore, TF2 also significantly decreased total mineral ash, including all measured inorganic compounds, except the fecal osmolytes sodium and potassium, which were increased and unchanged, respectively. These results suggest that the fiber enhanced mineral bioavailability in healthy dogs and dogs with managed CGE, relative to a food not enriched with fiber. This is consistent with the impact of pre-biotics included in foods [reviewed in Whisner and Castillo (44)], fiber supplementation in general (13), and our previous investigation of the same pre-biotic fiber bundle supplementation with CGE dogs in hydrolyzed foods enriched with either grain or meat (17). In contrast, although TF1 consumption reduced total fecal ash relative to TF2, the differences were less consistent than for TF1 vs. CF, with some minerals increased and others decreased. The reduced fecal moisture content associated with TF2 likely reflects the higher percentage of insoluble fiber included in the food vs. TF1.

### 4.2. Pre-biotic fiber bundle impacted microbial metabolism in the absence of major compositional changes

Our previous studies investigating the pre-biotic fiber bundle in different food backgrounds (17) and dogs with chronic large bowel diarrhea (16) showed evidence for a shift away from putrefactive toward saccharolytic microbiome activity using the same analytical tools and signatures investigated here in canine stool. In dogs consuming TF1 compared to CF, reductions in fecal total branched SCFA, ammonium, and MANOVA of amino acids and polyamines corroborated our previous observations supporting reduced proteolytic activity upon fiber ingestion. Furthermore, TF1 feeding resulted in reduced fecal abundance of the proteolytic genus *Clostridium* compared to both CF and TF2. Reduced microbial putrefactive activity in the lower gastrointestinal tract is largely considered a positive nutritional outcome for the host (45). Indeed, dogs with acute diarrhea present with increased levels of *Clostridium* (26, 46, 47), and the pathogenic bacterium *Clostridium difficile* is largely proteolytic (48). *Helicobacter* sp. are commonly found in canine feces, regardless of health status (49). High fiber foods, which would include TF1 and TF2, are known to negatively impact protein digestibility (17, 50). The pre-biotic fiber bundle appears to affect protein digestion by preventing fully digested amino acids from either reaching the colon or shifting microbes away from further metabolizing incompletely digested protein, which could explain why the universally decreased fecal amino acid signature was not fully consistent among dipeptides. The various fibers added to TF1 and TF2 clearly impact protein digestion and absorption differently. While TF2 contains slightly more protein by weight, consumption of TF1 resulted in higher fecal amino acid and dipeptide levels, as well as circulating amino acid levels. Despite the increased protein availability with TF1, microbial proteolysis was suppressed such that fecal ammonium levels were lower in TF1-fed dogs, while primary polyamine and branched SCFA levels were largely in line with those measured in the TF2 group.

Compared to CF, dogs consuming TF1 showed reduced signatures of saccharolytic metabolism, namely reduced straight SCFAs, possibly because TF1 contained 11% less total nitrogen-free extract, and possibly due to a reduction in residual resistant starch in TF1. In TF1, cornstarch was replaced with the fiber bundle, although it is not expected that cornstarch contributes more resistant starch given the extrusion conditions used to produce the food (23). Consuming the fiber bundle led to increased acetic acid but less propionic acid and lactate production. Intriguingly, the pH of feces from CF- and TF1-fed dogs were similar, even though CF resulted in higher overall SCFA and lactate levels, the largest contributors to fecal pH (51). Increased fecal levels of arabinose and ribulose/xylulose were consistent with our previous findings in dogs (16, 17) and cats (32, 52), and thus may represent a biochemical marker of ingesting this particular pre-biotic fiber.

Among the therapeutic foods, TF1 imparts a stronger fecal saccharolytic signature than TF2, including reduced pH and increased levels of lactate and monosaccharides, propionic and butanoic acids, and total straight SCFA. The higher fecal pH of dogs fed TF2 compared with TF1 could be attributed to a reduction in *Bifidobacterium* sp. abundance in combination with increased abundance of families Veillonellaceae, Enterobacteriaceae, as well as Clostridium and 02d06 genera within Clostridiaceae, all of which have been associated with elevated fecal pH in human infants (53). Members of the Ruminococcae family, including genus *Faecalibacterium*, generate butyrate from acetate, and their fecal abundances were higher among TF1-fed dogs compared to either CF or TF2. Lower levels of these taxa and the acetate producing *Bacteroidetes* were reported in dogs with acute diarrhea (46, 47, 54). Colonocytes consume microbiota-produced butanoic acid as their primary energy source through mitochondrial respiration (55). Both *Faecalibacterium* sp. and *Bacteroides* sp. play an important complementary role in maintaining colonic epithelial homeostasis by influencing the production of mucin glycans (56). Higher fecal lactate and straight SCFA levels, reduced pH, reduced ammonium, and reduced branched SCFAs have also been observed in dogs fed pomegranate pulp extract (51), while similar results were also reported in dogs consuming foods produced with higher levels of resistant starch (17, 57). Given that feces of TF2-fed dogs showed reduced butanoic acid and elevated pyrraline, a Maillard reaction product and marker of the thermal treatment of sugars in food processing (58), the TF2 food may have undergone more intense extrusion processing compared to TF1, in addition to including different pre-biotic fibers. Taken together, these results confirm that the composition and the preparation of TF1 and TF2 have differential effects on microbial fermentation and its subsequent beneficial post-biotic outputs in canine hindguts.

Mammalian hosts, including dogs, and their resident gut microbes readily exchange dietary tryptophan for a host of bioactive catabolites, such that these metabolites represent a noteworthy currency in assessing gut health, including barrier integrity and immune function, as well as systemic toxicity (8). Compared to control, TF1 consumption decreased most fecal monomeric amino acids, but not tryptophan. Integrated metabolomics and microbiome showed that enzymatic degradation of kynurenine by kynurenine-3-monooxygenase (K07130) function in feces was elevated, but downstream enzyme activity was reduced, possibly representing metabolic bottlenecks that ultimately resulted in decreases in 2-aminophenol, picolinate, and quinolinate. Suppression of most fecal nicotinamide adenine dinucleotide (NAD)-related metabolites could partly be due to reduced *de novo* NAD synthesis via quinolinate. Indole generation by tryptophanase is attributed to microbial activity, as mammals lack this enzyme functionality (59). Tryptophanase (K01667), as well as downstream nitrilase (K01501) and amidase (K01426) functions all were elevated in feces, while indole and indoleacetate levels were decreased and unchanged, respectively. These results imply that excess indole was quickly further catabolized to other indole species and/or indole and its catabolites were absorbed locally, as circulating levels were largely unaffected. By contrast, most fecal amino acid levels, including tryptophan, were higher with TF1 vs. TF2, which largely resulted in higher levels of other fecal TF1 tryptophan metabolites. The suppression of fecal quinolinate levels seen with the fiber bundle in TF1 was also evident vs. TF2; however, other NAD-related fecal metabolites were elevated with TF1 vs. TF2, suggesting the NAD salvage pathway more than compensated for any loss in *de novo* synthesis.

Analysis of circulating indole-related compounds provides clues into systemic health via gut microbiome metabolism. Compared to CF and TF2 consumption, TF1 consumption resulted in reduced serum levels of the indole sulfates 3-indoxyl sulfate, 5-hydroxyindole sulfate, and 7-hydroxyindole sulfate, as well as indolin-2-one (also known as oxindole), which was also lowest in feces of TF1-fed dogs, and these results were consistent with our previous study (16). Indoxyl sulfates are products of host-microbe biochemistry that result from the conversion of dietary tryptophan to indole by microbiota. Indole then enters the circulation and is hydroxylated and sulfated by the liver. Indoxyl sulfates are largely considered mammalian uremic toxins associated with acute and chronic kidney injury (60). By contrast, serum indoxyl glucuronide, another potential indole uremic toxin (61), was higher with TF1 vs. TF2 but not vs. CF. Indolin-2-one production may depend on multiple gut commensals and was implicated as a potent neurodepressive compound in a rat study (62). Others have reported that the inclusion of increasing levels of protein in pet foods fed to healthy dogs correlated with increasing serum levels of indole sulfates and fecal levels of indolin-2-one (7). Through a combination of reducing indole production and possible improved barrier function, consumption of the fiber bundle largely suppressed the production of several tryptophan-associated uremic toxins.

### 4.3. Fiber altered the anti-oxidative and anti-inflammatory capacity in the canine gastrointestinal tract

Metabolite components of the plant materials comprising the fiber bundle, as well as their microbe-metabolized post-biotics, were clearly evident in the feces of TF1-fed dogs, given the similar abundance differences between TF1 and both CF and TF2 fecal measurements. A previous study investigating TF1 as an interventional therapy in dogs with severe diarrhea also uniquely identified these plant-derived compounds in stool within 48 hours (30). In fact, several of the citrus pulp-derived phenolic (e.g., diosmetin, hesperidin, narirutin, phloretin, and sinensetin) and terpenoid (e.g., limonin and nomilin) components were presumably below their limits of detection in many of the samples from CF- and TF2-fed dogs, and comparison statistics were derived from imputation (data not shown). While the putative health benefits of phenolic phytonutrients are largely derived from *in vitro* studies, a growing body of clinical evidence in companion dogs corroborating the prebiotic, antioxidant, and immunomodulatory mechanisms of these benefits is emerging [reviewed extensively in Tanprasertsuk et al. (63)]. The inclusion of diverse phenolic bound fibers in dog food and their associated bioactive effects in canine hindguts may impart additional metabolic benefits for the host.

Post-biotics arising from microbiota-directed catabolism often are more bioavailable than their parent phenolic compounds, and thus may more strongly potentiate disease modification (25, 64). The gut microbiota readily converts the flavanone poncirin to ponciretin, and while both compounds attenuated chemically induced colitis and associated inflammation *in vivo*, ponciretin demonstrated more pronounced inflammasome inhibition *in vitro* (65). Here, only TF1-fed dogs showed increased ponciretin in feces; however, the presence of both compounds in citrus and absence of poncerin means we cannot definitively associate its presence in the TF1 group with microbiota metabolism. Similarly, the flavanone hesperetin is more bioactive than its glycoside parent compound hesperidin in its anti-inflammatory and radical scavenging properties (64). Hesperetin supplementation reduced cytokine secretion and improved epithelial barrier integrity in a chemically induced colitis model (66). The feces of TF1-fed dogs uniquely contained abundant hesperidin and hesperetin, yet the microbiome-directed contribution of the latter is unclear. Finally, orange juice rich in the flavanones hesperidin, narirutin, and their metabolites hesperetin and naringenin was shown in an *ex vivo* human feces fermentation study to act as a positive prebiotic, decreasing ammonium production and stimulating butyric, acetic, and propionic SCFA production and increasing antioxidant activity while affecting microbiota characteristics (67). The increase of ponciretin and hesperetin levels in the feces of dogs fed TF1 corroborates with the increased abundance levels of *Bacteroides* sp. and *Fusobacterium* sp. due to their potential alpha 1- rhamnosidase and beta-glucosidase activity in converting poncirin and hesperidin into ponciritin and hesperetin, respectively (68).

In a previous clinical study of TF1, the fiber-associated phenolics and their post-biotics were hypothesized to contribute to the resolution of chronic diarrhea resolution (16, 30). A recent murine study found significant, dose-dependent alleviation of colitis upon ingestion of eriodictyol, which was enriched in stools of TF1-fed dogs (69). A similar study investigating phloretin, which was also abundant in TF1-fed dog feces, reported the flavanone ameliorated colitis disease through a mechanism involving the regulation of the gut microbiota, including inflammatory modulation, restoration of barrier integrity, and increased anti-oxidative capacity (70). Phloretin may represent an intermediate post-biotic of flavonoid (e.g., apigenin and naringenin) microbial metabolism to 3-(4-hydoxyphenyl)-propionate (71). The gut microbiota can further metabolize the A and B rings of flavonoids into different stable phenolic acid end products. B-ring products include 3,4-dihydroxybenzoate and 4-hydroxybenzoate, while 2,4,6-trihydroxybenzoate can be derived from the A ring. TF1 consumption resulted in the fecal enrichment of 2,4,6-trihydroxybenzoate and 3,4-dihydroxybenzoate compared to both CF and TF1, and the fecal enrichment of 3-(4-hydroxyphenyl)-propionate and 4-hydroxybenzoate compared to TF2. The flavonoid post-biotic 2,4,6-trihydroxybenzoate was recently shown to have potent anti-colorectal cancer activity *in vitro* through inhibition of cyclin dependent kinase (72). Collectively, these results suggest that the citrus-derived phenolics and their post-biotics provided by the pre-biotic fiber bundle may have beneficial effects on the gastrointestinal health of dogs.

The lignans enterodiol and enterolactone and the isoflavone equol are bioactive phytoestrogens generated in mammals via the gut microbial metabolism of plant polyphenols (73, 74) and have been shown to provide anti-inflammatory, antioxidant, and free radical scavenging properties for the host (75). The TF1 ingredient flaxseed includes high levels of the lignan secoisolariciresinol diglucoside, which is metabolized by gut microbes to secoisolariciresinol and enterodiol. All three metabolites were uniquely enriched in stools of TF1-fed dogs in this study and in previous studies (17, 18). Consumption of TF1 compared to either CF or TF2, however, resulted in similar fecal levels of enterolactone and its parent lignan matairesinol, suggesting flaxseed largely contributes to microfloral production of enterodiol. While the soy isoflavones daidzein and genistein were present at higher levels in the stool of TF2-fed dogs compared to TF1-fed dogs and measured in all test samples, equol and glycitein levels were comparable among the therapeutic foods. In our previous study that switched dogs from various foods to TF1, all four isoflavones were significantly reduced (16). Given that the foods in this study appear to have trace soy-based ingredients, differences in downstream equol present in stools may have been negligible.

Dietary fiber is known to have profound effects on lipid digestion and absorption, depending on the composition, viscosity, and other physicochemical properties of the fiber (76). All three study foods contained a roughly equivalent percent weight of fish oil, a rich source of n3 PUFAs. TF1 also contained flaxseed, which likely contributed to elevated fecal levels of linolenate and linolenate-containing fatty acids in the TF1-fed group relative to the CF- or TF2-fed groups. Consumption of the pre-biotic fiber bundle in TF1 appeared to enhance lipolysis and mediate absorption of its free fatty acid substituents, resulting in higher levels of fecal monoacylglycerols and lysophospholipids, fecal and circulating free n3 and n6 PUFAs, and medium- and long-chain free fatty acids, while decreasing fecal levels of non-linolenate-containing diacylglycerols. Unfortunately, triacylglycerides were not measured in the metabolomic analysis, limiting the extent to which the dietary effects of the fiber bundle on lipolysis could be investigated. Among the therapeutic foods, fecal and serum n3 PUFAs as a class were higher in TF1-fed dogs, while circulating n6 PUFAs were lower, suggesting an improved n3/n6 ratio in healthy dogs consuming TF1, a finding consistent with the results of our previous intervention study in dogs with active large bowel diarrhea (16). Targeted MS/MS analysis would confirm such ratios. Serum levels of the inflammatory mediating eicosanoid lipid classes after TF1 consumption previously had reflected changes in their n3 and n6 precursors; however, in this study, these levels were similar regardless of diet, which is likely a reflection of the absence of active, uncontrolled disease.

Targeting the endocannabinoid system and related NAE biochemicals represents an emerging opportunity to impact gastrointestinal health, given their roles in mediating inflammation, gut motility, barrier function, and microbiota composition. While technically only AEA (also known as anandamide) and 2-arachidonoylglycerol (not measured here in feces) are endocannabinoid receptor agonists, the additional NAEs are non-selectively generated alongside AEA by the same enzymatic pathways, usually in higher yields, and are also implicated in gastrointestinal health and disease (77). The broad reductions in fecal NAEs with consumption of the pre-biotic fiber bundle corroborate our previous reports and appear independent of the disease status of the subjects (16, 17). Among the two fiber-containing foods tested, TF2 showed considerably higher fecal levels of AEA and other N-acylated amino acids, for example N-taurine- and N-serine-linked species, suggesting the phenomenon may be unique to the fiber blend in TF1 and not reflective of fiber supplementation overall. Investigations of NAE levels vary by gastrointestinal disease state, but broadly speaking, AEA and other NAEs are often inversely related to beneficial outcomes, whereas increased AEA and reduced palmitoylethanolamide and oleoylethanolamide have been associated with increased food intake, an increased risk of metabolic syndrome, and reduced barrier integrity (78). In a diet-driven obesity murine model, an activated endocannabinoid system influenced by the gut microbiome promoted barrier permeability and treatment with a pre-biotic restored AEA levels and barrier function (79). It is tempting to speculate that the novel pre-biotic fiber bundle modulates NAE generation and/or degradation to some degree, given the consistent and near-universally lower fecal levels associated with its intake; however, additional research is needed to support such a conclusion.

Sphingolipids and the enzymes involved in their metabolism modulate cell proliferation and immunity through mechanisms that can involve the gut microbiota (80). Obtained primarily from animal dietary sources as SM, most digestion occurs in the small intestine, which harbors the enzyme alkaline sphingomyelinase (alk-SMase), a potent degrader of SM to ceramide. Ceramidases hydrolyze ceramide to sphingosine, which is readily absorbed (81). Thus, circulating SM and ceramide are synthesized by the host and are unrelated to their respective levels in feces, which would reflect undigested sphingolipid. Consumption of the pre-biotic fiber bundle largely suppressed fecal and circulating sphingolipid levels compared to CF, with the exception of fecal SMs and serum ceramides, for which mixed directionality was observed. Since these foods presumably contained equivalent dietary sphingolipids, the data could reflect efficient alk-SMase and ceramidase activity and/or improved absorption by host tissues, as most sphingolipids were reduced in circulation. Gene expression or proteomics experiments may shed additional light on the fiber's modulation of sphingolipid metabolism. Alk-SMase activity correlates with improvements in inflammatory bowel disease and can be activated by probiotics in humans and mice (80). TF1 consumption largely resulted in higher fecal but lower serum levels of sphingolipids than TF2, including ceramides, but it did not affect fecal markers of sphingolipid synthesis (e.g., 3-ketosphinganine and sphinganine) or serum SM. TF1 may have contained higher dietary SM or TF2 may have improved sphingolipid absorption, given the similarities in markers of sphingolipid synthesis between groups and the generally higher sphingolipid levels in TF2.

### 4.4. Possible colonic mucosal layer restructuring upon pre-biotic fiber consumption

Phospholipids contribute to a hydrophobic barrier separating colonocytes from resident microbes, a critical function of the colonic mucosal layer. The phospholipid composition of the canine hindgut roughly follows its distribution along the entire gastrointestinal tract; PC and PE constitute approximately two thirds of the bulk, with ~10% each of SM, PI, and PS, and <5% lysophosphatidyl choline (82). In cases of inflammatory bowel disease in humans (83) and in dogs (84), irregularities in phospholipid levels at the tissue level and systemically have been documented. In fact, delayed release PC has been investigated as therapy for human ulcerative colitis (85).

Consumption of TF1 largely suppressed fecal PC, PE, SM, and lysophospholipids in dogs compared to CF and TF2, and while circulating phospholipids were also impacted, an overall decrease was evident in only SM and lysophospholipids. These results were more pronounced than those in our previous study in dogs with chronic large bowel diarrhea, in which PC and SM were significantly decreased in feces but were generally increased in serum (16). Taking both studies into account and acknowledging that the present study design controlled for environmental and breed variations, it appears the pre-biotic fiber bundle reduces phospholipid elimination in stool, regardless of disease state, while its impact on circulating phospholipids is more disease dependent. A study of circulating metabolites in children with ulcerative colitis or Crohn's disease also found irregularities in phospholipids vs. controls, with differing signatures associated with the specific inflammatory bowel disease (86). Interestingly, that study identified that the abundance of a single ceramide, LCER (18:1/16:0), could differentiate Crohn's disease from ulcerative colitis and that the level of serum LCER (18:1/1:0) was higher in Crohn's disease or ulcerative colitis than in controls. In our current study, fecal LCER (18:1/16:0) levels were lower, while serum levels were unchanged in TF1 vs. CF. In our previous intervention study, LCER levels increased in serum and were not measured in feces (16). Finally, n3 PUFA fatty acids (including those found in fish oil) are known to integrate into intestinal epithelial cells *in vivo* and have been associated with gut barrier and inflammatory protection (87). TF1 increased fecal and circulating n3 and n6 levels, but it is unknown whether gut epithelial tissue assimilated these protective lipids into membranes.

Two major factors limited the scope of this study. First, TF2 lacked a proper positive control, and since it was a commercially available food, we could not develop an appropriate control food for TF2 lacking its fiber supplement, as we did for TF1. We therefore limited our analysis to the effect of the fiber bundle under investigation (i.e., TF1 vs. CF) and the therapeutic foods themselves (i.e., TF1 vs. TF2). Thorough statistical analysis of the metabolomics data indicated the absence of period effects, which prompted us to combine the wash-in and washout feedings as controls. Second, the metabolomics measurements are quantitative but not absolute (i.e., do not provide concentrations), meaning we cannot properly compute metabolite ratios (e.g., n3/n6) or determine whether metabolite levels reached physiologically relevant concentrations. We therefore treated this study as exploratory and largely focused on metabolite pathway changes in our interpretation.

## 5. Conclusion

In summary, we identified gut microbiome structure and metabolism signatures associated with pre-biotic fiber supplementation in dogs. In our previous study investigating the impact of TF1 on the clinical resolution of chronic large bowel diarrhea, we inferred changes from baseline in dogs with active disease (16, 30). In this study, we largely corroborated those findings by comparing TF1 to a control food lacking the novel pre-biotic fiber bundle and to a different pre-biotic fiber-supplemented food, TF2, and further extended the results of the previous trial to both healthy dogs and dogs with controlled chronic gastroenteritis/enteritis. We demonstrated that consumption of the polyphenol-rich pre-biotic fiber blend in TF1 resulted in distinct fecal and circulating metabolomes from CF and TF2-fed dogs. While both TF1 and TF2 improved stool quality, TF1 feeding uniquely promoted microbial saccharolytic and post-biotic metabolism, while suppressing putrefactive processes, and further altered fecal and circulating lipid metabolism with positive implications for gut function and inflammation. This suggests TF1 is effective in both the early and continuing stages of veterinary management of gastroenteritis and demonstrates the value of comprehensive microbiome and metabolomic profiling in understanding how fiber interventions contribute to pet gastrointestinal health.

## Data availability statement

The raw data supporting the conclusions of this article will be made available by the authors, without undue reservation. The raw sequence data presented in this study are deposited in the National Center for Biotechnology and Information (NCBI) repository, accession number PRJNA918369. The data can be found here: https://www.ncbi.nlm.nih.gov/bioproject/?term=prjna918369.

## Ethics statement

The animal study was reviewed and approved by Institutional Animal Care and Use Committee (CP693a) of Hill's Pet Nutrition, Inc.

## Author contributions

DF, MJ, SW, and KG participated in the design and execution of the study. All authors participated in the data analysis and interpretation, drafted and revised the manuscript, and endorse the content of the work. All authors contributed to the article and approved the submitted version.
