# Supplementary material for: Adding a polyphenol-rich fiber bundle to food impacts the gastrointestinal microbiome and metabolome in dogs
Source: Front Vet Sci. 2023 Jan 20;9:1039032. doi: 10.3389/fvets.2022.1039032 (PMC9896628; doi:10.3389/fvets.2022.1039032)
Supplement: Supplementary file 1 [file Data_Sheet_1.PDF]

Supplemental Material 1

Canine Fiber Study Protocol

Study: Canine Fiber Study (94753)

Objective:

Determine effect of feeding a therapeutic food with a proprietary blend of soluble and insoluble fibers and botanicals to a population of healthy dogs and well-controlled dogs diagnosed with Inflammatory Bowel Disease (IBD). We are interested in measuring changes to the intestinal microflora, fecal nutrient composition, fecal pH, and post biotic short chain fatty acids production as measured in blood and feces. We are also interested in the effect of the dietary treatment on blood and fecal metabolomics such as inflammatory biomarkers and polyphenols.

Study Design:

This is a randomized controlled study of 20 healthy dogs and 20 dogs diagnosed with IBD. Each population of dogs was randomized into two treatment groups with case control matched pairs. All dogs were fed a scientific control food from day 1 - 28 of the study. Each population of dogs was randomized and fed one of two therapeutic diets from day 29 – 56 of the study. All dogs were fed a scientific control food from day 57 - 84 of the study. Each population of dogs was crossed-over and fed the other therapeutic food from day 84 – 112. Blood and fecal specimens will be collected on days 29, 56, 84, and 112.

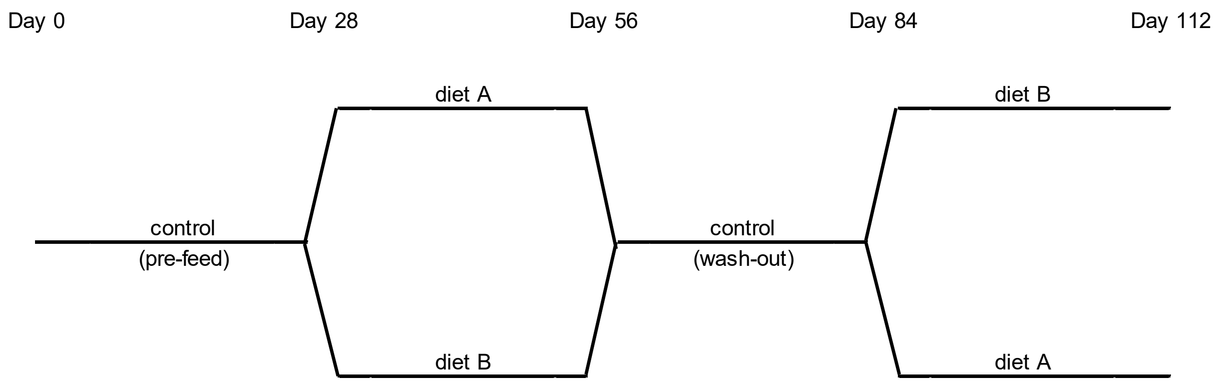

Study initiation: August 31, 2016

Study completion: December 20, 2016

Specimens: 160 blood and 160 fecal

Targeted ship date: December 21, 2016

Budget year: 2016

Supplementary Material 2. Fecal ash content analysis

Food	CF (n=78)	TF1 (n=39)	TF2 (n=39)	TF1 vs TF2	CF vs TF1
Ash component	Mean ± SE (ppm)	Mean ± SE (ppm)	Mean ± SE (ppm)	Difference ± SE (ppm)	Difference ± SE (ppm)
Calcium	63800 ± 1076	39000 ± 1360	36400 ± 1090	2700 ± 1262*	-25000 ± 1607***
Copper	89 ± 1.7	55 ± 2.1	45 ± 1.7	11 ± 2.3***	-34 ± 2.5***
Iron	2130 ± 37	1250 ± 49	1290 ± 23	-40 ± 28	-890 ± 59***
Magnesium	4039 ± 81	2820 ± 108	2400 ± 112	400 ± 122*	-1200 ± 117***
Manganese	219.5 ± 3.2	153 ± 5.5	285 ± 7.3	-130 ± 10***	-67 ± 5.9***
Phosphorus	35760 ± 631	19250 ± 771	21100 ± 566	-1800 ± 592*	-16500 ± 992***
Potassium	3220 ± 157	3010 ± 192	2700 ± 135	300 ± 122*	-200 ± 127
Sodium	2270 ± 231	3500 ± 270	3100 ± 233	400 ± 210	1200 ± 248***
Zinc	1558 ± 27	950 ± 32	750 ± 22	190 ± 25***	-610 ± 44***

*P<0.05; **P<0.001; ***P<0.0001

Supplementary Material 3. Fecal microbiome taxaonomy data

Selected taxonomy lineage at family and genera level showed significance ($P < 0.05$) were listed, as well as all phyla. Cells filled in green and red indicate increase and decrease in the dogs fed TF1, respectively. "ns" denotes taxa which were not significant for the test indicated, but were for the other test.

Bacterial taxonomy lineage	CF vs TF1		TF2 vs TF1	
	Estimate	Pr_BH	Estimate	Pr_BH
**Phylum**				
Actinobacteria	0.201	0.057	-0.117	0.415
Bacteroidetes	-0.249	0.203	0.032	0.917
Firmicutes	-0.014	0.808	0.005	0.917
Fusobacteria	0.214	0.010	-0.367	<0.001
Proteobacteria	-0.337	0.051	-0.063	0.782
**Family**				
Bacteroidaceae [p__Bacteroidetes; c__Bacteroidia; o__Bacteroidales]	0.623	0.018	ns	
Coriobacteriaceae [p__Actinobacteria; c__Coriobacteriia; o__Coriobacteriales]	ns		-0.482	0.001
Corynebacteriaceae [p__Actinobacteria; c__Actinobacteria; o__Actinomycetales]	ns		-0.356	0.012
Desulfovibrionaceae [p__Proteobacteria; c__Deltaproteobacteria; o__Desulfovibrionales]	-0.921	0.002	ns	
Enterobacteriaceae [p__Proteobacteria; c__Gammaproteobacteria; o__Enterobacteriales]	0.529	<0.001	-0.496	0.006
Erysipelotrichaceae [p__Firmicutes; c__Erysipelotrichi; o__Erysipelotrichales]	0.623	0.013	ns	
Fusobacteriaceae [p__Fusobacteria; c__Fusobacteriia; o__Fusobacteriales]	0.212	0.012	-0.374	<0.001
Helicobacteraceae [p__Proteobacteria; c__Epsilonproteobacteria; o__Campylobacterales]	ns		1.226	<0.001
Lactobacillaceae [p__Firmicutes; c__Bacilli; o__Lactobacillales]	-0.472	0.032	ns	
Mogibacteriaceae [p__Firmicutes; c__Clostridia; o__Clostridiales]	0.749	<0.001	ns	
Mycobacteriaceae [p__Actinobacteria; c__Actinobacteria; o__Actinomycetales]	0.625	0.029	ns	
Paraprevotellaceae [p__Bacteroidetes; c__Bacteroidia; o__Bacteroidales]	-0.397	0.026	ns	
Peptostreptococcaceae [p__Firmicutes; c__Clostridia; o__Clostridiales]	0.531	<0.001	ns	
Porphyromonadaceae [p__Bacteroidetes; c__Bacteroidia; o__Bacteroidales]	-0.813	<0.001	ns	
Pseudomonadaceae [p__Proteobacteria; c__Gammaproteobacteria; o__Pseudomonadales]	-0.942	<0.001	-0.525	0.011
Ruminococcaceae [p__Firmicutes; c__Clostridia; o__Clostridiales]	0.753	<0.001	0.469	<0.001
Turicibacteraceae [p__Firmicutes; c__Bacilli; o__Turicibacterales]	0.450	0.002	ns	
Veillonellaceae [p__Firmicutes; c__Clostridia; o__Clostridiales]	0.181	0.048	-0.276	0.013
**Genera**				
Anaerovibrio [p__Firmicutes; c__Clostridia; o__Clostridiales; f__Veillonellaceae]	-0.698	0.001	-0.800	0.006
Bacteroides [p__Bacteroidetes; c__Bacteroidia; o__Bacteroidales; f__Bacteroidaceae]	0.490	0.047	ns	
Bifidobacterium [p__Actinobacteria; c__Actinobacteria; o__Bifidobacteriales; f__Bifidobacteriaceae]	ns		0.410	0.054
Blautia [p__Firmicutes; c__Clostridia; o__Clostridiales; f__Lachnospiraceae]	ns		0.559	0.004
Clostridium [p__Firmicutes; c__Clostridia; o__Clostridiales; f__Clostridiaceae]	-0.485	0.007	-0.668	0.001
Collinsella [p__Actinobacteria; c__Coriobacteriia; o__Coriobacteriales; f__Coriobacteriaceae]	ns		-0.613	0.010
Corynebacterium [p__Actinobacteria; c__Actinobacteria; o__Actinomycetales; f__Corynebacteriaceae]	ns		-0.356	0.012
Desulfovibrio [p__Proteobacteria; c__Deltaproteobacteria; o__Desulfovibrionales; f__Desulfovibrionaceae]	-0.926	0.001	ns	
Dialister [p__Firmicutes; c__Clostridia; o__Clostridiales; f__Veillonellaceae]	ns		1.247	<0.001
Enterococcus [p__Firmicutes; c__Bacilli; o__Lactobacillales; f__Enterococcaceae]	-0.522	0.020	ns	
Epulopiscium [p__Firmicutes; c__Clostridia; o__Clostridiales; f__Lachnospiraceae]	0.523	0.002	ns	
Faecalibacterium [p__Firmicutes; c__Clostridia; o__Clostridiales; f__Ruminococcaceae]	0.783	<0.001	0.776	<0.001
Helicobacter [p__Proteobacteria; c__Epsilonproteobacteria; o__Campylobacterales; f__Helicobacteraceae]	ns		1.566	<0.001
Lactobacillus [p__Firmicutes; c__Bacilli; o__Lactobacillales; f__Lactobacillaceae]	-0.472	0.034	ns	
Megamonas [Firmicutes; c__Clostridia; o__Clostridiales; f__Veillonellaceae]	0.344	0.007	ns	
Megasphaera [p__Firmicutes; c__Clostridia; o__Clostridiales; f__Veillonellaceae]	ns		-0.818	<0.001
Mycobacterium[p__Actinobacteria; c__Actinobacteria; o__Actinomycetales; f__Mycobacteriaceae]	0.625	0.031	ns	
Paraprevotella [p__Bacteroidetes; c__Bacteroidia; o__Bacteroidales; f__Paraprevotellaceae]	-1.246	<0.001	ns	
Phascolarctobacterium [p__Firmicutes; c__Clostridia; o__Clostridiales; f__Veillonellaceae]	ns		-0.459	0.003
Pseudomonas [p__Proteobacteria; c__Gammaproteobacteria; o__Pseudomonadales; f__Pseudomonadacea	-0.969	<0.001	-0.547	0.010
Succinivibrio [p__Proteobacteria; c__Gammaproteobacteria; o__Aeromonadales; f__Succinivibrionaceae]	ns		-0.530	0.048
Turicibacter [p__Firmicutes; c__Bacilli; o__Turicibacterales; f__Turicibacteraceae]	0.450	0.002	ns	
Veillonella [p__Firmicutes; c__Clostridia; o__Clostridiales; f__Veillonellaceae]	0.309	0.054	-0.611	<0.001
02d06 [p__Firmicutes; c__Clostridia; o__Clostridiales; f__Clostridiaceae]	-0.542	0.027	-1.157	<0.001

Supplementary Material 4. Principal Component Analysis summary for metabolomics data

Principal Component	Eigenvalue		Proportion (%)		Cumulative Proportion (%)	
	Feces	Serum	Feces	Serum	Feces	Serum
**1**	89.6	73.5	12.4	10.6	12.4	10.6
**2**	71.6	52.4	9.9	7.5	22.3	18.1
**3**	50.6	45.9	7	6.6	29.3	24.7
**4**	41.4	44.5	5.7	6.4	35	31.1
**5**	32.8	31.3	4.5	4..5	39.5	35.6
**6**	30.3	28.3	4.2	4.2	43.7	39.7
**7**	25.9	22.9	3.6	3.3	47.3	43
**8**	19.4	19.8	2.7	2.9	49.9	45.9
**9**	17.4	19	2.4	2.7	52.3	48.6

Supplementary Material 5. Full fecal metabolomics data

For each test, green and red indicate metabolites within the pathway significantly increased or decreased, respectively, while yellow indicates the pa[illegible] significantly altered but overall directionality was inconclusive.

FECES	TF1 vs CF				TF1 vs TF2			
Group and Metabolite	MANOVA	Difference	SE	Pr > |t|	MANOVA	Difference	SE	Pr > |t|
**Alkaloids**	<.0001	.	.	.	<.0001	.	.	.
DIMBOA	.	-0.37	0.05	<.0001	.	0.48	0.03	<.0001
1-methyl-beta-carboline-3-carboxylic acid	.	-0.93	0.09	<.0001	.	0.19	0.05	0.0006
deoxymugineic acid	.	-0.01	0.03	0.8580	.	0.31	0.04	<.0001
dipicolinate	.	-1.31	0.72	0.0776	.	0.15	0.54	0.7884
ergothioneine	.	0.02	0.19	0.9045	.	-0.09	0.20	0.6485
nicotianamine	.	-0.39	0.11	0.0013	.	0.25	0.30	0.4126
piperidine	.	0.78	0.15	<.0001	.	1.45	0.18	<.0001
pyrraline	.	-0.01	0.06	0.8274	.	-4.83	0.33	<.0001
solanidine	.	-9.38	7.21	0.2035	.	-0.14	0.10	0.1832
stachydrine	.	2.90	0.27	<.0001	.	3.18	0.28	<.0001
**Amino Acids**	<.0001	.	.	.	<.0001	.	.	.
alanine	.	-0.12	0.06	0.0486	.	0.31	0.05	<.0001
arginine	.	0.25	0.17	0.1426	.	1.09	0.16	<.0001
asparagine	.	-0.08	0.29	0.7906	.	0.73	0.29	0.0155
aspartate	.	0.12	0.10	0.2173	.	0.23	0.15	0.1296
cysteine	.	-0.41	0.08	<.0001	.	0.12	0.04	0.0029
glutamate	.	-0.03	0.14	0.8041	.	0.39	0.10	0.0004
glutamine	.	-0.08	0.11	0.4749	.	0.56	0.08	<.0001
glycine	.	-0.32	0.10	0.0041	.	0.19	0.07	0.0067
histidine	.	-0.12	0.11	0.2748	.	0.41	0.09	<.0001
isoleucine	.	-0.20	0.08	0.0195	.	0.33	0.06	<.0001
leucine	.	-0.07	0.07	0.3316	.	0.39	0.07	<.0001
lysine	.	-0.17	0.06	0.0041	.	0.26	0.05	<.0001
methionine	.	-0.21	0.06	0.0017	.	0.31	0.05	<.0001
phenylalanine	.	-0.12	0.08	0.1303	.	0.35	0.07	<.0001
proline	.	-0.18	0.07	0.0113	.	0.08	0.05	0.0991
serine	.	-0.02	0.09	0.8518	.	0.42	0.08	<.0001
taurine	.	0.50	0.32	0.1243	.	-0.32	0.47	0.4969
threonine	.	-0.34	0.10	0.0014	.	0.35	0.07	<.0001
tryptophan	.	-0.09	0.09	0.3066	.	0.46	0.07	<.0001
tyrosine	.	0.02	0.06	0.7295	.	0.36	0.06	<.0001
valine	.	-0.22	0.09	0.0230	.	0.38	0.08	<.0001
**Benzoate Metabolism**	<.0001	.	.	.	<.0001	.	.	.
2,4,6-trihydroxybenzoate	.	4.68	0.29	<.0001	.	4.78	0.43	<.0001
3-(2-hydroxyphenyl)propionate	.	-0.23	0.08	0.0086	.	0.02	0.07	0.7439
3-(3-hydroxyphenyl)propionate	.	0.08	0.09	0.3702	.	0.42	0.10	0.0002
3,4-dihydroxybenzoate	.	1.05	0.20	<.0001	.	0.76	0.26	0.0064
3-(4-hydroxyphenyl)propionate	.	0.18	0.13	0.1652	.	0.84	0.15	<.0001
4-hydroxybenzoate	.	0.11	0.14	0.4211	.	0.67	0.11	<.0001
3-phenylpropionate (hydrocinnamate)	.	-0.11	0.11	0.2873	.	0.21	0.11	0.0641
**Carbohydrate Metabolism**	<.0001	.	.	.	<.0001	.	.	.
arabinose	.	1.79	0.24	<.0001	.	1.55	0.25	<.0001
arabitol/xylitol	.	1.32	0.73	0.0765	.	1.72	1.05	0.1103
arabonate/xylonate	.	-0.07	0.21	0.7337	.	0.05	0.22	0.8266
erythritol	.	-0.38	0.12	0.0038	.	0.29	0.14	0.0488
erythronate*	.	-0.19	0.21	0.3690	.	0.46	0.11	0.0001
erythrose	.	-0.24	0.17	0.1750	.	0.16	0.17	0.3425

fructose	.	-0.20	0.11	0.0658	.	-0.20	0.11	0.0693
fucose	.	-0.11	0.24	0.6462	.	0.35	0.23	0.1369
galactitol (dulcitol)	.	0.05	0.21	0.8081	.	0.85	0.25	0.0016
galactonate	.	0.14	0.16	0.3962	.	0.43	0.14	0.0047
galacturonate	.	2.39	0.26	<.0001	.	1.09	0.26	0.0002
glucose	.	-0.10	0.11	0.3746	.	0.53	0.10	<.0001
glucuronate	.	-0.04	0.10	0.7190	.	0.07	0.21	0.7480
glycerate	.	0.04	0.17	0.8328	.	0.43	0.17	0.0153
lactate	.	-1.88	0.68	0.0094	.	0.66	0.17	0.0006
maltose	.	-0.15	0.19	0.4459	.	0.94	0.19	<.0001
mannitol/sorbitol	.	-0.42	0.19	0.0345	.	0.22	0.10	0.0369
mannose	.	-0.16	0.10	0.1132	.	-0.58	0.11	<.0001
pyruvate	.	0.01	0.18	0.9653	.	0.74	0.24	0.0033
ribose	.	-0.24	0.06	0.0001	.	-0.11	0.09	0.2048
ribulose/xylulose	.	0.54	0.11	<.0001	.	0.42	0.12	0.0019
xylose	.	-0.81	0.08	<.0001	.	-1.30	0.11	<.0001

**Collagen Metabolism**	0.0001	.	.	.	<.0001	.	.	.
5-hydroxylysine	.	-0.31	0.12	0.0112	.	-1.86	0.65	0.0067
5-(galactosylhydroxy)-L-lysine	.	-0.93	0.21	0.0001	.	-0.76	0.10	<.0001
hydroxyproline	.	-0.09	0.05	0.0825	.	-0.58	0.09	<.0001

**Diacylglycerol**	<.0001	.	.	.	<.0001	.	.	.
diacylglycerol (16:1/18:2 [2], 16:0/18:3 [1])*	.	0.04	0.07	0.5556	.	-1.11	0.14	<.0001
palmitoyl-oleoyl-glycerol (16:0/18:1) [2]*	.	-0.48	0.08	<.0001	.	-0.70	0.12	<.0001
oleoyl-oleoyl-glycerol (18:1/18:1) [1]*	.	-0.30	0.17	0.0754	.	-0.24	0.16	0.1469
oleoyl-oleoyl-glycerol (18:1/18:1) [2]*	.	-0.49	0.11	0.0001	.	-0.17	0.08	0.0398
palmitoyl-linolenoyl-glycerol (16:0/18:3) [2]*	.	1.67	0.25	<.0001	.	1.97	0.29	<.0001
oleoyl-linolenoyl-glycerol (18:1/18:3) [2]*	.	1.95	0.23	<.0001	.	2.25	0.31	<.0001
linoleoyl-linolenoyl-glycerol (18:2/18:3) [1]*	.	1.08	0.19	<.0001	.	1.53	0.21	<.0001
linoleoyl-linolenoyl-glycerol (18:2/18:3) [2]*	.	0.96	0.17	<.0001	.	1.49	0.19	<.0001
linolenoyl-linolenoyl-glycerol (18:3/18:3) [1]*	.	5.60	0.74	<.0001	.	6.25	0.80	<.0001
linolenoyl-linolenoyl-glycerol (18:3/18:3) [2]*	.	7.56	0.84	<.0001	.	8.74	1.00	<.0001
palmitoyl-arachidonoyl-glycerol (16:0/20:4) [2]*	.	-0.12	0.09	0.2014	.	-0.87	0.13	<.0001
oleoyl-arachidonoyl-glycerol (18:1/20:4) [2]*	.	0.02	0.09	0.7951	.	-1.14	0.16	<.0001
linoleoyl-arachidonoyl-glycerol (18:2/20:4) [2]*	.	-0.05	0.09	0.5986	.	-0.64	0.12	<.0001
palmitoyl-linoleoyl-glycerol (16:0/18:2) [1]*	.	-0.47	0.09	<.0001	.	-0.24	0.08	0.0061
palmitoyl-linoleoyl-glycerol (16:0/18:2) [2]*	.	-0.57	0.06	<.0001	.	-0.21	0.06	0.0029
palmitoleoyl-linoleoyl-glycerol (16:1/18:2) [1]*	.	-0.34	0.09	0.0004	.	-1.64	0.18	<.0001
stearoyl-linoleoyl-glycerol (18:0/18:2) [2]*	.	-0.14	0.11	0.1964	.	-0.51	0.14	0.0008
oleoyl-linoleoyl-glycerol (18:1/18:2) [1]	.	-0.38	0.13	0.0065	.	0.05	0.08	0.5356
oleoyl-linoleoyl-glycerol (18:1/18:2) [2]	.	-0.50	0.09	<.0001	.	0.03	0.06	0.6512
linoleoyl-linoleoyl-glycerol (18:2/18:2) [1]*	.	-0.53	0.12	0.0001	.	0.29	0.09	0.0017
linoleoyl-linoleoyl-glycerol (18:2/18:2) [2]*	.	-1.13	0.19	<.0001	.	0.22	0.11	0.0425

**Dipeptides**	<.0001	.	.	.	<.0001	.	.	.
alanylleucine	.	0.05	0.08	0.5385	.	0.45	0.09	<.0001
glutaminylleucine	.	0.49	0.10	<.0001	.	0.93	0.09	<.0001
glycylisoleucine	.	0.24	0.07	0.0024	.	0.11	0.12	0.3745
glycylleucine	.	-0.17	0.09	0.0587	.	0.33	0.07	0.0001
glycylvaline	.	-0.17	0.08	0.0442	.	0.35	0.07	<.0001
isoleucylglycine	.	-0.37	0.11	0.0013	.	-0.05	0.08	0.5095
leucylalanine	.	0.32	0.09	0.0005	.	0.46	0.11	0.0003
leucylglutamine*	.	0.36	0.08	<.0001	.	0.50	0.09	<.0001
leucylglycine	.	-0.13	0.08	0.1245	.	0.27	0.07	0.0008
lysylleucine	.	-0.06	0.07	0.4084	.	0.12	0.09	0.1878
phenylalanylalanine	.	0.01	0.10	0.9150	.	0.36	0.11	0.0019

phenylalanylglycine	.	-0.27	0.08	0.0012	.	0.32	0.07	<.0001
threonylphenylalanine	.	0.58	0.11	<.0001	.	0.76	0.11	<.0001
tryptophylglycine	.	-0.21	0.10	0.0415	.	0.40	0.11	0.0007
tyrosylglycine	.	-0.23	0.07	0.0018	.	0.31	0.07	0.0001
valylglutamine	.	0.15	0.06	0.0298	.	0.34	0.10	0.0013
valylglycine	.	-0.32	0.09	0.0015	.	0.14	0.08	0.0661
valylleucine	.	0.16	0.07	0.0343	.	0.54	0.10	<.0001
**Endocannabinoids**	<.0001	.	.	.	<.0001	.	.	.
N-arachidonoyltaurine	.	0.18	0.25	0.4643	.	-0.03	0.20	0.8909
N-oleoylserine	.	-0.21	0.11	0.0539	.	-1.06	0.23	0.0001
N-oleoyltaurine	.	0.44	0.26	0.0990	.	-0.63	0.22	0.0068
N-palmitoylserine	.	-1.02	0.17	<.0001	.	-0.09	0.11	0.4155
N-palmitoyltaurine	.	0.05	0.14	0.7305	.	-1.22	0.24	<.0001
N-stearoyltaurine	.	-0.08	0.05	0.0954	.	-1.11	0.23	<.0001
arachidonoyl ethanolamide	.	-0.53	0.08	<.0001	.	-2.84	0.38	<.0001
arachidoyl ethanolamide (20:0)*	.	-0.59	0.10	<.0001	.	-0.62	0.09	<.0001
behenoyl ethanolamide (22:0)*	.	-0.71	0.13	<.0001	.	-0.65	0.10	<.0001
dihomo-linolenoyl ethanolamide	.	-0.41	0.07	<.0001	.	-2.48	0.28	<.0001
erucoyl ethanolamide (22:1)*	.	-0.64	0.11	<.0001	.	-0.58	0.07	<.0001
lignoceroyl ethanolamide (24:0)*	.	-0.49	0.14	0.0008	.	-0.08	0.07	0.2743
linoleoyl ethanolamide	.	-0.56	0.11	<.0001	.	0.00	0.11	0.9904
margaroyl ethanolamide*	.	-0.73	0.10	<.0001	.	-0.46	0.08	<.0001
myristoyl ethanolamide	.	-0.47	0.08	<.0001	.	-0.44	0.08	<.0001
nervonoyl ethanolamide (24:1)*	.	-0.51	0.13	0.0004	.	-0.23	0.08	0.0036
oleoyl ethanolamide	.	-0.66	0.09	<.0001	.	-0.58	0.12	<.0001
palmitoleoyl ethanolamide*	.	-0.55	0.09	<.0001	.	-1.47	0.24	<.0001
palmitoyl ethanolamide	.	-0.56	0.07	<.0001	.	-0.74	0.10	<.0001
stearoyl ethanolamide	.	-0.49	0.07	<.0001	.	-0.93	0.07	<.0001
**Free Fatty Acid - Polyunsaturated n3 Fatty Acids**	<.0001	.	.	.	<.0001	.	.	.
hexadecatrienoate (16:3n3)	.	-0.08	0.05	0.1187	.	-0.24	0.05	<.0001
heneicosapentaenoate (21:5n3)	.	0.87	0.22	0.0004	.	0.24	0.23	0.3029
linolenate (18:3n3 or 3n6)	.	4.81	0.47	<.0001	.	4.64	0.47	<.0001
nisinate (24:6n3)	.	0.56	0.38	0.1466	.	1.10	0.43	0.0158
docosahexaenoate (DHA; 22:6n3)	.	1.45	0.39	0.0008	.	1.31	0.41	0.0027
docosapentaenoate (DPA; 22:5n3)	.	0.65	0.25	0.0124	.	0.41	0.33	0.2198
eicosapentaenoate (EPA; 20:5n3)	.	0.94	0.22	0.0002	.	1.00	0.25	0.0003
stearidonate (18:4n3)	.	0.24	0.08	0.0041	.	0.20	0.08	0.0189
**Free Fatty Acids - Polyunsaturated n6 Fatty Acids**	0.0184	.	.	.	<.0001	.	.	.
arachidonate (20:4n6)	.	0.57	0.28	0.0489	.	0.38	0.27	0.1673
dihomolinoleate (20:2n6)	.	0.08	0.18	0.6388	.	-0.36	0.28	0.1965
docosadienoate (22:2n6)	.	0.30	0.19	0.1225	.	-0.41	0.34	0.2386
hexadecadienoate (16:2n6)	.	0.21	0.06	0.0018	.	-0.52	0.07	<.0001
linoleate (18:2n6)	.	0.51	0.13	0.0002	.	0.21	0.15	0.1736
docosapentaenoate (n6 DPA; 22:5n6)	.	0.43	0.14	0.0038	.	-0.04	0.20	0.8348
**Free Fatty Acids - Short, Medium, and Long Chain Fatty Acid**	<.0001	.	.	.	<.0001	.	.	.
10-nonadecenoate (19:1n9)	.	0.52	0.12	0.0001	.	0.05	0.20	0.8159
caprate (10:0)	.	-0.17	0.11	0.1500	.	0.02	0.04	0.6989
caproate (6:0)	.	-4.62	1.24	0.0007	.	0.67	0.25	0.0100
caprylate (8:0)	.	-0.18	0.31	0.5592	.	0.22	0.10	0.0279
erucate (22:1n9)	.	0.32	0.12	0.0099	.	-0.88	0.41	0.0405
eicosenoate (20:1n9 or 1n11)	.	0.37	0.13	0.0075	.	-0.13	0.30	0.6797
myristoleate (14:1n5)	.	0.00	0.06	0.9365	.	-1.43	0.09	<.0001
oleate/vaccenate (18:1)	.	0.55	0.12	<.0001	.	-0.32	0.22	0.1464

palmitoleate (16:1n7)	.	0.40	0.09	0.0001	.	-1.82	0.25	<.0001
valerate (5:0)	.	-2.77	0.59	<.0001	.	0.68	0.18	0.0007

**Hemoglobin Metabolism**	<.0001	.	.	.	0.1841	.	.	.
D-urobilin	.	-0.57	0.22	0.0127	.	-0.04	0.21	0.8621
I-urobilinogen	.	-1.07	0.31	0.0014	.	-0.09	0.27	0.7470
L-urobilin	.	-0.27	0.12	0.0311	.	0.08	0.11	0.5005
bilirubin	.	-1.35	0.28	<.0001	.	0.06	0.16	0.6798
bilirubin (E,E)*	.	-0.69	0.14	<.0001	.	0.20	0.12	0.1052
bilirubin (E,Z or Z,E)*	.	-0.32	0.17	0.0615	.	0.08	0.11	0.4787
biliverdin	.	-0.37	0.11	0.0015	.	0.07	0.12	0.5632

**Linolenate Metabolism**	<.0001	.	.	.	<.0001	.	.	.
1-linolenoylglycerol (18:3)	.	6.98	1.52	<.0001	.	6.81	1.57	0.0001
1-linoleoyl-2-linolenoyl-digalactosylglycerol (18:2/18:3)*	.	-0.03	0.10	0.7693	.	0.32	0.08	0.0006
1,2-dilinolenoyl-digalactosylglycerol (18:3/18:3)	.	-0.21	0.32	0.5079	.	-1.10	1.30	0.3996
linolenate (18:3n3 or 3n6)	.	4.81	0.47	<.0001	.	4.64	0.47	<.0001
palmitoyl-linolenoyl-glycerol (16:0/18:3) [2]*	.	1.67	0.25	<.0001	.	1.97	0.29	<.0001
oleoyl-linolenoyl-glycerol (18:1/18:3) [2]*	.	1.95	0.23	<.0001	.	2.25	0.31	<.0001
linoleoyl-linolenoyl-glycerol (18:2/18:3) [1]*	.	1.08	0.19	<.0001	.	1.53	0.21	<.0001
linoleoyl-linolenoyl-glycerol (18:2/18:3) [2]*	.	0.96	0.17	<.0001	.	1.49	0.19	<.0001
linolenoyl-linolenoyl-glycerol (18:3/18:3) [1]*	.	5.60	0.74	<.0001	.	6.25	0.80	<.0001
linolenoyl-linolenoyl-glycerol (18:3/18:3) [2]*	.	7.56	0.84	<.0001	.	8.74	1.00	<.0001
dihomolinolenate (20:3n3 or 3n6)	.	0.18	0.19	0.3286	.	-0.30	0.30	0.3178
1-linoleoyl-2-linolenoyl-galactosylglycerol (18:2/18:3)*	.	-0.57	0.58	0.3317	.	-0.47	0.42	0.2717
1,2-dilinolenoyl-galactosylglycerol (18:3/18:3)*	.	-0.74	0.94	0.4353	.	-3.80	4.19	0.3714
**Monoacylglycerol**	<.0001	.	.	.	<.0001	.	.	.
1-linolenoylglycerol (18:3)	.	6.98	1.52	<.0001	.	6.81	1.57	0.0001
1-linoleoylglycerol (18:2)	.	0.22	0.22	0.3183	.	-0.12	0.21	0.5733
1-oleoylglycerol (18:1)	.	0.35	0.14	0.0199	.	-0.74	0.25	0.0054
1-palmitoleoylglycerol (16:1)*	.	0.51	0.18	0.0077	.	-3.59	0.58	<.0001
2-linoleoylglycerol (18:2)	.	0.50	0.29	0.0929	.	-0.10	0.42	0.8083
2-oleoylglycerol (18:1)	.	0.53	0.19	0.0081	.	-0.89	0.36	0.0176
1-docosahexaenoylglycerol (22:6)	.	0.86	0.29	0.0060	.	-0.45	0.36	0.2252
**Nicotinate and Nicotinamide Metabolism**	<.0001	.	.	.	<.0001	.	.	.
1-methylnicotinamide	.	-0.39	0.48	0.4249	.	1.28	0.51	0.0167
6-hydroxynicotinate	.	-0.34	0.08	0.0002	.	0.45	0.10	<.0001
trigonelline (N'-methylnicotinate)	.	0.25	0.22	0.2549	.	1.33	0.13	<.0001
nicotinamide	.	0.82	0.28	0.0058	.	2.04	0.33	<.0001
nicotinamide riboside	.	-0.55	0.09	<.0001	.	0.21	0.07	0.0036
nicotinate	.	-0.26	0.05	<.0001	.	0.17	0.04	0.0002
nicotinate ribonucleoside	.	-0.24	0.22	0.2954	.	0.97	0.19	<.0001
quinolinate	.	-0.60	0.26	0.0282	.	-0.44	0.10	0.0002
**Oxidized compounds**	0.0042	.	.	.	0.0052	.	.	.
3-methyl-2-oxobutyrate	.	-0.56	0.20	0.0071	.	-0.04	0.20	0.8451
3-methyl-2-oxovalerate	.	-0.52	0.19	0.0079	.	-0.20	0.24	0.4029
4-methyl-2-oxopentanoate	.	-0.31	0.15	0.0465	.	-0.02	0.25	0.9359
4-hydroxyphenylpyruvate	.	-0.20	0.14	0.1614	.	-0.15	0.19	0.4574
phenylpyruvate	.	-0.42	0.17	0.0176	.	-0.08	0.25	0.7556
pyruvate	.	0.01	0.18	0.9653	.	0.74	0.24	0.0033
**Phenolic Compounds**	<.0001	.	.	.	<.0001	.	.	.
apigenin	.	1.06	0.16	<.0001	.	1.21	0.20	<.0001
chrysoeriol	.	2.13	0.37	<.0001	.	2.69	0.42	<.0001

daidzein	.	-0.15	0.15	0.3133	.	-0.41	0.09	0.0001
dihydrokaempferol	.	2.28	0.49	<.0001	.	2.73	0.53	<.0001
diosmetin	.	1.51	0.17	<.0001	.	1.50	0.17	<.0001
eriodictyol	.	35.17	5.70	<.0001	.	35.21	5.77	<.0001
ferulate	.	0.00	0.13	0.9700	.	0.09	0.13	0.4713
feruloylputrescine	.	2.01	2.89	0.4898	.	3.40	2.89	0.2466
genistein	.	-0.19	0.24	0.4406	.	-0.32	0.12	0.0101
glycitein	.	-0.96	0.88	0.2866	.	0.28	0.36	0.4425
hesperidin	.	2.28	0.40	<.0001	.	2.33	0.39	<.0001
secoisolariciresinol diglucoside	.	7.22	0.76	<.0001	.	7.22	0.84	<.0001
matairesinol	.	-0.30	0.45	0.5124	.	0.31	0.19	0.1117
narirutin	.	2.13	0.57	0.0006	.	2.18	0.56	0.0004
phloretin	.	1.10	0.17	<.0001	.	1.09	0.16	<.0001
sinapate	.	-0.87	0.47	0.0726	.	0.53	0.14	0.0007
sinensetin	.	1.09	0.09	<.0001	.	1.09	0.09	<.0001
syringic acid	.	-0.74	0.38	0.0583	.	1.05	0.30	0.0011
tyrosol	.	0.16	0.07	0.0252	.	0.70	0.09	<.0001
vanillate	.	0.13	0.26	0.6254	.	1.06	0.21	<.0001
**Phospholipids - Lysophospholipid**	<.0001	.	.	.	<.0001	.	.	.
1-linoleoyl-GPC (18:2)	.	0.09	0.07	0.1800	.	0.45	0.12	0.0007
1-linoleoyl-GPE (18:2)*	.	-0.08	0.06	0.1631	.	0.40	0.08	<.0001
1-oleoyl-GPC (18:1)	.	0.17	0.09	0.0581	.	0.55	0.13	0.0002
1-oleoyl-GPE (18:1)	.	0.25	0.05	<.0001	.	0.40	0.06	<.0001
1-palmitoyl-GPC (16:0)	.	0.10	0.11	0.4088	.	0.49	0.10	<.0001
1-palmitoyl-GPE (16:0)	.	0.33	0.07	<.0001	.	0.26	0.07	0.0009
1-palmitoyl-GPG (16:0)*	.	0.29	0.10	0.0090	.	0.55	0.11	<.0001
1-palmitoyl-GPI* (16:0)	.	0.62	0.17	0.0010	.	0.78	0.22	0.0012
1-stearoyl-GPC (18:0)	.	0.51	0.27	0.0694	.	0.99	0.24	0.0002
1-stearoyl-GPE (18:0)	.	0.16	0.32	0.6211	.	1.15	0.22	<.0001
1-stearoyl-GPG (18:0)	.	0.67	0.16	0.0001	.	0.23	0.22	0.2961
1-stearoyl-GPI (18:0)	.	1.03	0.31	0.0017	.	1.09	0.26	0.0001
1-stearoyl-GPS (18:0)*	.	0.40	0.37	0.2796	.	1.28	0.23	<.0001
**Phospholipids - Phosphatidylcholine (PC)**	<.0001	.	.	.	<.0001	.	.	.
1,2-dilinoleoyl-GPC (18:2/18:2)	.	-0.76	0.08	<.0001	.	-0.08	0.06	0.1579
1,2-dipalmitoyl-GPC (16:0/16:0)	.	-0.47	0.09	<.0001	.	-0.25	0.06	0.0001
1-palmitoyl-2-arachidonoyl-GPC (16:0/20:4n6)	.	-0.15	0.08	0.0910	.	-0.59	0.08	<.0001
1-stearoyl-2-arachidonoyl-GPC (18:0/20:4)	.	-0.12	0.10	0.2363	.	-0.38	0.08	0.0001
1-stearoyl-2-oleoyl-GPC (18:0/18:1)	.	-0.35	0.09	0.0005	.	-0.36	0.06	<.0001
1-palmitoyl-2-oleoyl-GPC (16:0/18:1)	.	-0.45	0.06	<.0001	.	-0.29	0.05	<.0001
1-palmitoyl-2-docosahexaenoyl-GPC (16:0/22:6)	.	0.07	0.10	0.4810	.	-0.49	0.10	<.0001
1-palmitoyl-2-stearoyl-GPC (16:0/18:0)	.	-0.55	0.11	<.0001	.	0.13	0.08	0.0814
1-palmitoyl-2-palmitoleoyl-GPC (16:0/16:1)*	.	-0.46	0.09	<.0001	.	-0.66	0.09	<.0001
1-palmitoyl-2-linoleoyl-GPC (16:0/18:2)	.	-0.62	0.07	<.0001	.	-0.30	0.05	<.0001
1-oleoyl-2-linoleoyl-GPC (18:1/18:2)*	.	-0.79	0.09	<.0001	.	0.01	0.05	0.7761
**Phospholipids - Phosphatidylethanolamine (PE)**	<.0001	.	.	.	0.0002	.	.	.
1,2-dilinoleoyl-GPE (18:2/18:2)*	.	-0.66	0.12	<.0001	.	-0.01	0.08	0.9433
1,2-dipalmitoyl-GPE (16:0/16:0)*	.	-0.54	0.09	<.0001	.	-0.22	0.09	0.0206
1-palmitoyl-2-arachidonoyl-GPE (16:0/20:4)*	.	-0.30	0.08	0.0007	.	-0.42	0.15	0.0076
1-palmitoyl-2-oleoyl-GPE (16:0/18:1)	.	-0.49	0.10	<.0001	.	-0.48	0.10	<.0001
1-palmitoyl-2-linoleoyl-GPE (16:0/18:2)	.	-0.71	0.09	<.0001	.	-0.22	0.10	0.0276
1-oleoyl-2-linoleoyl-GPE (18:1/18:2)*	.	-0.50	0.09	<.0001	.	0.00	0.07	0.9446
**Phospholipid Metabolism**	<.0001	.	.	.	<.0001	.	.	.
choline	.	-0.21	0.08	0.0130	.	0.37	0.05	<.0001

glycerophosphoserine*	.	0.18	0.21	0.4058	.	0.63	0.15	0.0002
glycerophosphoinositol*	.	0.01	0.14	0.9185	.	0.54	0.09	<.0001
glycerophosphorylcholine (GPC)	.	-0.22	0.18	0.2183	.	0.13	0.08	0.1375
phosphocholine	.	-0.29	0.23	0.2173	.	0.45	0.33	0.1857
glycerophosphoethanolamine	.	0.16	0.05	0.0042	.	0.45	0.05	<.0001
trimethylamine N-oxide	.	-0.28	0.08	0.0014	.	0.13	0.07	0.0746
**Polyamines**	<.0001	.	.	.	<.0001	.	.	.
4-acetamidobutanoate	.	-0.45	0.14	0.0024	.	0.77	0.15	<.0001
N1,N12-diacetylspermine	.	-2.74	0.31	<.0001	.	0.12	0.08	0.1464
(N(1) + N(8))-acetylspermidine	.	-0.99	0.07	<.0001	.	-0.18	0.08	0.0325
N(1)-acetylspermine	.	-3.46	0.54	<.0001	.	-0.19	0.14	0.1778
N-acetyl-cadaverine	.	-0.66	0.21	0.0029	.	0.51	0.15	0.0013
N-acetylputrescine	.	-0.30	0.10	0.0033	.	0.31	0.10	0.0052
agmatine	.	0.31	0.56	0.5871	.	0.76	0.65	0.2463
cadaverine	.	-1.59	0.27	<.0001	.	0.02	0.13	0.9065
carboxyethyl-GABA	.	-0.25	0.06	0.0001	.	-0.27	0.05	<.0001
5-methylthioadenosine (MTA)	.	-0.72	0.20	0.0007	.	0.51	0.11	<.0001
ornithine	.	0.13	0.18	0.4588	.	0.70	0.17	0.0002
putrescine	.	-0.60	0.08	<.0001	.	0.03	0.07	0.6753
spermidine	.	-2.01	0.14	<.0001	.	-0.30	0.07	0.0001
**Postbiotics**	<.0001	.	.	.	<.0001	.	.	.
2-piperidinone	.	-0.13	0.05	0.0081	.	0.09	0.05	0.0952
3-dehydroshikimate	.	0.05	0.14	0.7403	.	0.39	0.13	0.0039
4-hydroxycinnamate	.	0.95	0.10	<.0001	.	0.44	0.11	0.0003
diaminopimelate	.	-0.30	0.14	0.0365	.	0.11	0.09	0.2075
enterodiol	.	3.22	0.37	<.0001	.	3.36	0.44	<.0001
enterolactone	.	0.85	1.26	0.5018	.	1.84	1.41	0.2013
equol	.	-0.47	1.59	0.7666	.	1.83	1.38	0.1907
hesperetin	.	580.51	67.21	<.0001	.	578.28	67.07	<.0001
naringenin	.	43.92	4.83	<.0001	.	44.13	5.07	<.0001
ponciretin	.	141.54	14.27	<.0001	.	140.68	14.38	<.0001
secoisolariciresinol	.	22.97	3.45	<.0001	.	24.03	3.42	<.0001
beta-guanidinopropanoate	.	0.09	0.05	0.0730	.	-0.43	0.06	<.0001
**Primary bile acids**	<.0001	.	.	.	0.0149	.	.	.
taurochenodeoxycholate	.	0.58	0.31	0.0622	.	-1.62	0.58	0.0085
chenodeoxycholate	.	-0.15	0.06	0.0225	.	-0.01	0.02	0.4765
cholate	.	-1.78	0.56	0.0029	.	-0.10	0.39	0.7921
glycocholate	.	0.46	0.38	0.2387	.	-0.31	0.61	0.6122
7alpha-hydroxycholestenone	.	-0.58	0.12	<.0001	.	-0.21	0.07	0.0065
7-hydroxycholesterol (alpha or beta)	.	-0.55	0.10	<.0001	.	-0.21	0.12	0.0951
3b-hydroxy-5-cholenoic acid	.	-0.53	0.08	<.0001	.	0.02	0.05	0.6568
glycochenodeoxycholate	.	0.02	0.13	0.8878	.	-0.07	0.16	0.6437
tauro-beta-muricholate	.	-0.23	0.36	0.5192	.	-4.45	3.99	0.2715
taurocholate	.	1.84	0.47	0.0005	.	-3.41	3.32	0.3118
**Reduced compounds**	0.0085	.	.	.	<.0001	.	.	.
lactate	.	-1.88	0.68	0.0094	.	0.66	0.17	0.0006
alpha-hydroxyisocaproate	.	-0.94	0.43	0.0339	.	1.37	0.31	0.0001
alpha-hydroxyisovalerate	.	-0.70	0.31	0.0326	.	1.24	0.20	<.0001
phenyllactate (PLA)	.	-1.26	0.38	0.0018	.	1.07	0.27	0.0003
3-(4-hydroxyphenyl)lactate (HPLA)	.	-1.69	0.69	0.0189	.	1.81	0.65	0.0079
2-hydroxy-3-methylvalerate	.	-1.20	0.42	0.0064	.	1.21	0.25	<.0001
**Secondary Bile Acids**	0.0002	.	.	.	0.0069	.	.	.

12-dehydrocholate	.	-0.73	0.35	0.0425	.	0.22	0.35	0.5410
3-dehydrocholate	.	-0.18	0.20	0.3763	.	-0.02	0.20	0.9334
6-oxolithocholate	.	-0.76	0.23	0.0027	.	0.06	0.12	0.6072
7-ketodeoxycholate	.	-0.80	0.35	0.0264	.	0.27	0.33	0.4137
7-ketolithocholate	.	-0.76	0.18	0.0002	.	0.11	0.17	0.5291
dehydrolithocholate	.	-0.28	0.21	0.1840	.	0.21	0.34	0.5333
deoxycholate	.	-0.19	0.06	0.0042	.	0.04	0.06	0.5010
glycodeoxycholate	.	-0.25	0.14	0.0829	.	-0.25	0.19	0.2065
glycohyodeoxycholate	.	-0.03	0.15	0.8288	.	0.11	0.09	0.2613
hyocholate	.	-0.55	0.07	<.0001	.	0.25	0.04	<.0001
isohyodeoxycholate	.	-0.90	0.23	0.0004	.	0.10	0.04	0.0274
isoursodeoxycholate	.	-0.37	0.08	0.0001	.	0.17	0.11	0.1471
lithocholate	.	-0.23	0.07	0.0020	.	0.04	0.07	0.6118
taurodeoxycholate	.	0.10	0.37	0.7853	.	-2.52	1.57	0.1171
taurolithocholate	.	0.27	0.28	0.3349	.	-1.16	0.94	0.2265
tauroursodeoxycholate	.	0.63	0.22	0.0062	.	-0.07	0.45	0.8813
ursocholate	.	-0.19	0.36	0.6035	.	0.39	0.37	0.3011
ursodeoxycholate	.	0.07	0.14	0.6234	.	0.38	0.26	0.1471
**Sphingolipids - Ceramides**	<.0001	.	.	.	<.0001	.	.	.
ceramide (d18:1/20:0, d16:1/22:0, d20:1/18:0)*	.	-0.25	0.08	0.0033	.	-0.10	0.08	0.2553
ceramide (d18:1/17:0, d17:1/18:0)*	.	-0.41	0.11	0.0006	.	0.41	0.10	0.0003
ceramide (d18:2/24:1, d18:1/24:2)*	.	-0.17	0.10	0.0778	.	0.49	0.12	0.0002
N-palmitoyl-sphingosine (d18:1/16:0)	.	-0.23	0.07	0.0013	.	0.29	0.07	0.0004
N-stearoyl-sphingosine (d18:1/18:0)*	.	-0.41	0.13	0.0028	.	-0.30	0.11	0.0107
N-oleoyl-sphingosine (d18:1/18:1)*	.	-0.28	0.29	0.3445	.	-0.23	0.28	0.4107
N-(2-hydroxypalmitoyl)-sphingosine (d18:1/16:0(2OH))	.	-0.66	0.16	0.0002	.	-0.15	0.14	0.2919
**Sphingolipids - Dihydroceramides**	0.0046	.	.	.	0.0004	.	.	.
N-palmitoyl-sphinganine (d18:0/16:0)	.	-0.23	0.09	0.0142	.	0.17	0.08	0.0423
N-stearoyl-sphinganine (d18:0/18:0)*	.	-0.41	0.12	0.0018	.	-0.24	0.15	0.1149
**Sphingolipids - Dihydrosphingomyelins**	0.0686	.	.	.	0.0016	.	.	.
sphingomyelin (d18:0/18:0, d19:0/17:0)*	.	0.07	0.08	0.4133	.	0.25	0.11	0.0252
palmitoyl dihydrosphingomyelin (d18:0/16:0)*	.	-0.06	0.09	0.5203	.	0.47	0.12	0.0006
**Sphingolipids - Hexosylceramides (HCER)**	<.0001	.	.	.	<.0001	.	.	.
glycosyl ceramide (d18:1/20:0, d16:1/22:0)*	.	-0.31	0.07	0.0001	.	0.18	0.09	0.0500
glycosyl-N-palmitoyl-sphingosine (d18:1/16:0)	.	-0.23	0.06	0.0002	.	0.54	0.08	<.0001
glycosyl-N-stearoyl-sphingosine (d18:1/18:0)	.	-0.57	0.07	<.0001	.	-0.13	0.12	0.2512
glycosyl-N-(2-hydroxynervonoyl)-sphingosine (d18:1/24:1(2OH))	.	-0.32	0.10	0.0024	.	0.59	0.10	<.0001
**Sphingolipids - Lactosylceramides (LCER)**	<.0001	.	.	.	<.0001	.	.	.
lactosyl-N-palmitoyl-sphingosine (d18:1/16:0)	.	-0.44	0.09	<.0001	.	0.53	0.10	<.0001
**Sphingolipids - Sphingolipid Synthesis**	0.0019	.	.	.	0.3686	.	.	.
3-ketosphinganine	.	-0.37	0.10	0.0010	.	-0.24	0.13	0.0696
phytosphingosine	.	-0.59	0.18	0.0028	.	-0.21	0.16	0.1902
sphingadienine	.	-0.27	0.27	0.3262	.	-0.44	0.26	0.1005
sphinganine	.	-0.42	0.15	0.0072	.	-0.26	0.14	0.0714
**Sphingolipids - Sphingosines**	0.0144	.	.	.	<.0001	.	.	.
N-acetylsphingosine	.	-1.23	0.44	0.0084	.	-0.90	0.48	0.0655
eicosanoylsphingosine (d20:1)*	.	-0.70	0.21	0.0019	.	-0.57	0.17	0.0019
sphingosine	.	-0.40	0.12	0.0019	.	-0.37	0.11	0.0020
heptadecasphingosine (d17:1)	.	-0.60	0.24	0.0157	.	-0.10	0.19	0.5915
hexadecasphingosine (d16:1)*	.	-0.57	0.27	0.0433	.	-0.04	0.18	0.8050

**Sphingomyelins**	0.0003	.	.	.	0.0026	.	.	.
sphingomyelin (d18:1/17:0, d17:1/18:0, d19:1/16:0)	.	-0.18	0.16	0.2653	.	0.85	0.18	<.0001
palmitoyl sphingomyelin (d18:1/16:0)	.	-0.10	0.12	0.4217	.	0.69	0.16	0.0001
stearoyl sphingomyelin (d18:1/18:0)	.	0.00	0.15	0.9807	.	0.42	0.21	0.0521
behenoyl sphingomyelin (d18:1/22:0)*	.	0.25	0.17	0.1396	.	0.77	0.23	0.0015
tricosanoyl sphingomyelin (d18:1/23:0)*	.	0.32	0.16	0.0487	.	0.85	0.23	0.0007
lignoceroyl sphingomyelin (d18:1/24:0)	.	0.20	0.14	0.1531	.	0.84	0.21	0.0004
sphingomyelin (d18:1/20:0, d16:1/22:0)*	.	0.06	0.12	0.6461	.	0.64	0.18	0.0012
sphingomyelin (d18:1/24:1, d18:2/24:0)*	.	0.03	0.17	0.8521	.	0.61	0.22	0.0090
sphingomyelin (d18:2/24:1, d18:1/24:2)*	.	0.07	0.16	0.6846	.	0.88	0.25	0.0014
**Terpenoids**	<.0001	.	.	.	<.0001	.	.	.
beta-sitosterol	.	-0.41	0.04	<.0001	.	0.44	0.03	<.0001
campesterol	.	-0.42	0.04	<.0001	.	0.34	0.03	<.0001
carotene diol (1)	.	-0.34	0.05	<.0001	.	0.17	0.03	<.0001
carotene diol (2)	.	-0.48	0.07	<.0001	.	0.17	0.03	<.0001
carotene diol (3)	.	-0.52	0.08	<.0001	.	0.33	0.03	<.0001
ergosterol	.	-0.27	0.03	<.0001	.	-0.86	0.04	<.0001
lanosterol	.	-0.37	0.08	0.0001	.	0.05	0.07	0.4943
limonin	.	2.12	0.40	<.0001	.	2.10	0.39	<.0001
nomilin	.	1.96	0.29	<.0001	.	1.94	0.41	<.0001
pheophorbide A	.	0.81	0.36	0.0295	.	1.09	0.11	<.0001
stigmasterol	.	-0.47	0.04	<.0001	.	0.33	0.03	<.0001
**Tocopherol Metabolism**	<.0001	.	.	.	<.0001	.	.	.
alpha-tocopherol	.	-0.32	0.02	<.0001	.	-0.06	0.02	0.0136
alpha-tocotrienol	.	-0.38	0.08	<.0001	.	0.32	0.09	0.0017
delta-tocopherol	.	-0.18	0.03	<.0001	.	-0.37	0.04	<.0001
gamma-tocotrienol	.	-0.07	0.06	0.2360	.	-0.27	0.13	0.0468
alpha-tocopherol acetate	.	-0.65	0.20	0.0022	.	-0.16	0.12	0.1803
gamma-tocopherol/beta-tocopherol	.	-0.22	0.03	<.0001	.	-0.16	0.04	0.0007
**Tryptophan Metabolism - Indole Pathway**	<.0001	.	.	.	<.0001	.	.	.
2-oxindole-3-acetate	.	-0.72	0.07	<.0001	.	0.42	0.04	<.0001
3-hydroxyindolin-2-one	.	0.17	0.11	0.1354	.	-0.07	0.10	0.4769
3-indoxyl sulfate	.	-0.57	1.09	0.6060	.	-0.12	0.29	0.6906
indole	.	-0.47	0.18	0.0140	.	-0.09	0.16	0.5593
indoleacetate	.	-0.09	0.07	0.1857	.	0.67	0.08	<.0001
indoleacetylglutamine	.	-0.61	0.44	0.1759	.	0.48	0.16	0.0050
indoleacetylglycine	.	-2.08	1.28	0.1136	.	0.19	0.30	0.5322
indoleacrylate	.	0.03	0.14	0.8146	.	0.33	0.18	0.0689
indolelactate	.	-1.76	0.60	0.0055	.	1.41	0.44	0.0028
indolepropionate	.	-0.30	0.22	0.1904	.	0.82	0.18	0.0001
indolin-2-one	.	-1.21	0.27	0.0001	.	-0.41	0.14	0.0077
tryptamine	.	-0.48	0.15	0.0037	.	0.21	0.11	0.0572
**Tryptophan Metabolism - Kynurenine Pathway**	<.0001	.	.	.	<.0001	.	.	.
2-aminophenol	.	-0.39	0.08	<.0001	.	0.72	0.07	<.0001
anthranilate	.	-0.80	0.19	0.0001	.	0.27	0.12	0.0269
N-formylanthranilic acid	.	-0.16	0.06	0.0142	.	-0.28	0.08	0.0008
kynurenate	.	-3.73	2.80	0.1916	.	3.28	1.35	0.0204
kynurenine	.	0.06	0.05	0.1920	.	0.55	0.05	<.0001
picolinate	.	-0.62	0.08	<.0001	.	0.16	0.05	0.0036
quinolinate	.	-0.60	0.26	0.0282	.	-0.44	0.10	0.0002
xanthurenate	.	-1.48	2.42	0.5440	.	2.94	1.57	0.0690

**Tryptophan Metabolism - Serotonin Pathway**	0.0003	.	.	.	<.0001	.	.	.
5-hydroxyindoleacetate	.	-0.23	0.06	0.0012	.	0.26	0.08	0.0031
serotonin	.	-0.17	0.08	0.0382	.	0.34	0.07	<.0001

Supplementary Material 6. Full serum metabolomics data

For each test, green and red indicate metabolites within the pathway significantly increased or decreased, respectively, while yellow indicates the pathway was significantly altered but overall directionality was inconclusive.

SERUM	TF1 vs CF				TF1 vs TF2			
Group and Metabolite	MANOVA	Difference	SE	Pr > |t|	MANOVA	Difference	SE	Pr > |t|
**Amino Acids**	<.0001	.	.	.	<.0001	.	.	.
alanine	.	-0.14	0.03	0.0002	.	0.11	0.03	0.0005
arginine	.	-0.03	0.02	0.0512	.	-0.03	0.02	0.1697
asparagine	.	0.00	0.02	0.8097	.	0.00	0.02	0.9114
aspartate	.	-0.02	0.04	0.6121	.	0.14	0.04	0.0006
cysteine	.	-0.24	0.05	<.0001	.	-0.15	0.05	0.0106
glutamate	.	-0.01	0.02	0.7809	.	0.16	0.03	<.0001
glutamine	.	-0.02	0.02	0.1918	.	0.05	0.01	0.0008
glycine	.	-0.03	0.02	0.1326	.	-0.09	0.03	0.0008
histidine	.	-0.03	0.01	0.0332	.	0.02	0.01	0.0964
isoleucine	.	-0.01	0.02	0.5708	.	-0.04	0.02	0.0838
leucine	.	0.00	0.02	0.8377	.	0.02	0.02	0.2429
lysine	.	0.01	0.02	0.6266	.	0.07	0.02	0.0055
methionine	.	-0.05	0.02	0.0131	.	0.09	0.02	0.0001
phenylalanine	.	-0.05	0.02	0.0037	.	0.03	0.02	0.1050
proline	.	-0.09	0.02	0.0001	.	0.07	0.02	0.0066
serine	.	0.04	0.02	0.0950	.	-0.06	0.03	0.0247
taurine	.	-0.18	0.06	0.0079	.	-0.18	0.05	0.0008
threonine	.	-0.03	0.03	0.3138	.	0.12	0.04	0.0039
tryptophan	.	-0.11	0.02	<.0001	.	0.02	0.02	0.4998
tyrosine	.	-0.07	0.02	0.0057	.	0.08	0.03	0.0220
valine	.	-0.01	0.02	0.6807	.	-0.07	0.03	0.0123
**Carbohydrate Metabolism**	<.0001	.	.	.	<.0001	.	.	.
arabitol/xylitol	.	-0.04	0.04	0.2413	.	0.18	0.03	<.0001
arabonate/xylonate	.	-0.09	0.04	0.0357	.	0.04	0.05	0.4126
erythritol	.	-0.10	0.03	0.0008	.	0.17	0.02	<.0001
erythronate*	.	-0.06	0.02	0.0043	.	0.11	0.02	<.0001
fructose	.	-0.03	0.05	0.5739	.	-0.06	0.04	0.1245
glucose	.	-0.03	0.02	0.1043	.	-0.02	0.01	0.2308
glucuronate	.	0.12	0.05	0.0154	.	0.20	0.04	<.0001
glycerate	.	-0.07	0.03	0.0430	.	0.01	0.03	0.7910
lactate	.	-0.23	0.04	<.0001	.	0.12	0.04	0.0048
mannitol/sorbitol	.	0.07	0.08	0.3342	.	-0.09	0.13	0.4895
mannose	.	0.22	0.03	<.0001	.	-0.19	0.03	<.0001
pyruvate	.	-0.72	0.13	<.0001	.	0.08	0.09	0.3536
ribitol	.	0.00	0.03	0.9960	.	0.12	0.02	<.0001
ribose	.	-0.42	0.12	0.0010	.	0.27	0.07	0.0006
xylose	.	0.36	0.15	0.0186	.	0.23	0.16	0.1739
**Collagen Metabolism**	<.0001	.	.	.	<.0001	.	.	.
5-hydroxylysine	.	0.21	0.09	0.0307	.	-0.04	0.11	0.7301
hydroxyproline	.	-0.10	0.02	<.0001	.	-0.81	0.06	<.0001
prolylhydroxyproline	.	0.07	0.10	0.4399	.	0.05	0.09	0.5693
**Free Fatty Acids - Eicosanoids**	0.4516	.	.	.	0.8588	.	.	.
12-HETE	.	-1.71	1.08	0.1221	.	0.70	0.84	0.4102
12-HHTrE	.	-1.40	0.99	0.1656	.	0.63	0.76	0.4142
prostaglandin F2alpha	.	-0.23	0.16	0.1677	.	0.04	0.12	0.7572
thromboxane B2	.	-0.44	0.26	0.0968	.	0.10	0.17	0.5573

**Free Fatty Acids - Polyunsaturated n3 Fatty Acids**	<.0001	.	.	.	<.0001	.	.	.
docosatrienoate (22:3n3)	.	0.07	0.07	0.3465	.	-0.08	0.13	0.5079
hexadecatrienoate (16:3n3)	.	0.20	0.13	0.1511	.	0.15	0.16	0.3516
heneicosapentaenoate (21:5n3)	.	0.10	0.10	0.2923	.	0.06	0.17	0.7104
linolenate (18:3n3 or 3n6)	.	0.74	0.10	<.0001	.	0.69	0.14	<.0001
docosahexaenoate (DHA; 22:6n3)	.	0.08	0.04	0.0781	.	0.00	0.09	0.9747
docosapentaenoate (DPA; 22:5n3)	.	-0.09	0.05	0.0454	.	-0.15	0.10	0.1425
eicosapentaenoate (EPA; 20:5n3)	.	0.14	0.06	0.0251	.	0.32	0.11	0.0061
stearidonate (18:4n3)	.	0.19	0.09	0.0501	.	0.21	0.13	0.1309
**Free Fatty Acids - Polyunsaturated n6 Fatty Acids**	0.0009	.	.	.	<.0001	.	.	.
adrenate (22:4n6)	.	-0.01	0.05	0.7795	.	-0.20	0.07	0.0059
arachidonate (20:4n6)	.	-0.04	0.04	0.2292	.	-0.28	0.07	0.0002
dihomolinoleate (20:2n6)	.	0.08	0.05	0.1240	.	-0.02	0.10	0.8800
docosadienoate (22:2n6)	.	0.10	0.04	0.0174	.	-0.17	0.11	0.1289
docosatrienoate (22:3n6)*	.	0.02	0.08	0.7641	.	-0.77	0.13	<.0001
hexadecadienoate (16:2n6)	.	0.21	0.08	0.0084	.	-0.01	0.12	0.9440
linoleate (18:2n6)	.	0.16	0.04	0.0009	.	0.05	0.06	0.4401
docosapentaenoate (n6 DPA; 22:5n6)	.	-0.13	0.04	0.0031	.	-0.46	0.08	<.0001
**Free Fatty Acids - Short, Medium, and Long Chain Fatty Acic**	0.0012	.	.	.	<.0001	.	.	.
10-heptadecenoate (17:1n7)	.	0.21	0.07	0.0057	.	0.03	0.11	0.7734
10-nonadecenoate (19:1n9)	.	0.23	0.08	0.0072	.	0.02	0.12	0.8924
arachidate (20:0)	.	0.05	0.02	0.0019	.	-0.05	0.04	0.2951
caproate (6:0)	.	0.00	0.06	0.9467	.	-0.05	0.07	0.4792
erucate (22:1n9)	.	0.16	0.04	0.0002	.	-0.45	0.14	0.0034
heptanoate (7:0)	.	0.46	0.24	0.0628	.	0.35	0.30	0.2603
margarate (17:0)	.	0.06	0.04	0.1563	.	-0.03	0.08	0.7344
myristate (14:0)	.	0.09	0.05	0.0807	.	0.03	0.07	0.7319
myristoleate (14:1n5)	.	0.21	0.09	0.0228	.	0.04	0.09	0.6684
nervonate (24:1n9)*	.	0.09	0.03	0.0026	.	-0.37	0.14	0.0114
nonadecanoate (19:0)	.	0.13	0.03	0.0002	.	0.02	0.08	0.8492
oleate/vaccenate (18:1)	.	0.16	0.05	0.0011	.	-0.06	0.06	0.2999
palmitate (16:0)	.	0.07	0.02	0.0120	.	-0.09	0.04	0.0477
palmitoleate (16:1n7)	.	0.26	0.08	0.0022	.	-0.12	0.12	0.3120
pentadecanoate (15:0)	.	0.06	0.02	0.0066	.	-0.05	0.05	0.2809
stearate (18:0)	.	0.02	0.02	0.2264	.	-0.04	0.03	0.1356
valerate (5:0)	.	0.03	0.07	0.6963	.	0.09	0.07	0.1810
**Phospholipids - Lysophospholipid**	<.0001	.	.	.	<.0001	.	.	.
1-arachidonoyl-GPA (20:4)	.	-0.05	0.03	0.0944	.	-0.33	0.07	<.0001
1-arachidonoyl-GPC* (20:4)*	.	-0.05	0.03	0.1513	.	-0.26	0.04	<.0001
1-arachidonoyl-GPI* (20:4)*	.	0.07	0.06	0.2201	.	-0.17	0.07	0.0286
1-lignoceroyl-GPC (24:0)	.	-0.06	0.04	0.1348	.	0.23	0.06	0.0012
1-linolenoyl-GPC (18:3)*	.	0.92	0.06	<.0001	.	1.10	0.07	<.0001
1-linoleoyl-GPA (18:2)*	.	-0.03	0.04	0.4140	.	-0.02	0.06	0.7921
1-linoleoyl-GPC (18:2)	.	0.00	0.03	0.9086	.	-0.06	0.04	0.1256
1-linoleoyl-GPE (18:2)*	.	0.04	0.04	0.3490	.	0.06	0.04	0.1641
1-linoleoyl-GPG (18:2)*	.	-0.14	0.06	0.0409	.	-0.11	0.14	0.4526
1-linoleoyl-GPI* (18:2)*	.	0.04	0.08	0.6400	.	-0.18	0.15	0.2312
1-oleoyl-GPC (18:1)	.	0.00	0.03	0.9510	.	-0.15	0.03	<.0001
1-oleoyl-GPE (18:1)	.	0.05	0.04	0.1846	.	-0.02	0.03	0.5484
1-palmitoleoyl-GPC* (16:1)*	.	-0.08	0.03	0.0268	.	-0.18	0.04	<.0001
1-palmitoyl-GPA (16:0)	.	-0.11	0.04	0.0053	.	-0.26	0.09	0.0048
1-palmitoyl-GPC (16:0)	.	-0.12	0.03	<.0001	.	-0.27	0.03	<.0001
1-palmitoyl-GPE (16:0)	.	-0.13	0.03	0.0001	.	-0.22	0.04	<.0001
1-palmitoyl-GPI* (16:0)	.	-0.15	0.09	0.1176	.	-0.51	0.17	0.0043

1-stearoyl-GPC (18:0)	.	-0.04	0.02	0.0447	.	-0.08	0.03	0.0026
1-stearoyl-GPE (18:0)	.	-0.03	0.02	0.1660	.	-0.06	0.03	0.0462
1-stearoyl-GPG (18:0)	.	-0.12	0.10	0.2705	.	-0.05	0.15	0.7419
1-stearoyl-GPI (18:0)	.	0.08	0.06	0.1564	.	-0.02	0.08	0.7484
2-stearoyl-GPE (18:0)*	.	-0.03	0.04	0.4463	.	-0.15	0.10	0.1456
**Phospholipids - Phosphatidylcholine (PC)**	<.0001	.	.	.	<.0001	.	.	.
1,2-dilinoleoyl-GPC (18:2/18:2)	.	0.06	0.03	0.0793	.	0.12	0.04	0.0022
1,2-dipalmitoyl-GPC (16:0/16:0)	.	-0.16	0.02	<.0001	.	-0.15	0.02	<.0001
1-palmitoyl-2-arachidonoyl-GPC (16:0/20:4n6)	.	-0.06	0.02	0.0017	.	-0.27	0.02	<.0001
1-myristoyl-2-arachidonoyl-GPC (14:0/20:4)*	.	-0.04	0.03	0.2301	.	0.09	0.03	0.0114
1-stearoyl-2-arachidonoyl-GPC (18:0/20:4)	.	-0.01	0.02	0.6403	.	-0.11	0.02	<.0001
1-stearoyl-2-oleoyl-GPC (18:0/18:1)	.	-0.07	0.02	0.0013	.	-0.23	0.03	<.0001
1,2-dilinolenoyl-GPC (18:3/18:3)*	.	0.22	0.07	0.0025	.	0.51	0.07	<.0001
1-palmitoyl-2-oleoyl-GPC (16:0/18:1)	.	-0.09	0.02	<.0001	.	-0.37	0.02	<.0001
1-palmitoleoyl-2-linolenoyl-GPC (16:1/18:3)*	.	0.38	0.05	<.0001	.	0.62	0.05	<.0001
1-linoleoyl-2-linolenoyl-GPC (18:2/18:3)*	.	0.79	0.05	<.0001	.	1.16	0.07	<.0001
1-palmitoyl-2-docosahexaenoyl-GPC (16:0/22:6)	.	-0.10	0.03	0.0023	.	-0.19	0.04	<.0001
1-stearoyl-2-docosahexaenoyl-GPC (18:0/22:6)	.	-0.06	0.02	0.0050	.	-0.01	0.02	0.6139
1-oleoyl-2-docosahexaenoyl-GPC (18:1/22:6)*	.	0.13	0.03	0.0003	.	0.33	0.05	<.0001
1-palmitoyl-2-stearoyl-GPC (16:0/18:0)	.	-0.11	0.03	0.0002	.	-0.17	0.03	<.0001
1-palmitoyl-2-palmitoleoyl-GPC (16:0/16:1)*	.	-0.14	0.03	<.0001	.	-0.34	0.03	<.0001
1-palmitoyl-2-linoleoyl-GPC (16:0/18:2)	.	-0.07	0.01	<.0001	.	-0.20	0.01	<.0001
1-palmitoleoyl-2-linoleoyl-GPC (16:1/18:2)*	.	-0.06	0.03	0.0893	.	-0.12	0.03	0.0011
1-stearoyl-2-linoleoyl-GPC (18:0/18:2)*	.	0.00	0.01	0.7467	.	0.01	0.02	0.5316
1-myristoyl-2-palmitoyl-GPC (14:0/16:0)	.	-0.45	0.07	<.0001	.	0.11	0.03	0.0020
**Phospholipids - Phosphatidylethanolamine (PE)**	0.0001	.	.	.	<.0001	.	.	.
1-palmitoyl-2-arachidonoyl-GPE (16:0/20:4)*	.	-0.08	0.03	0.0199	.	-0.25	0.04	<.0001
1-stearoyl-2-arachidonoyl-GPE (18:0/20:4)	.	-0.01	0.03	0.6925	.	0.00	0.04	0.8902
1-stearoyl-2-oleoyl-GPE (18:0/18:1)	.	-0.04	0.04	0.2426	.	0.05	0.06	0.3610
1-palmitoyl-2-oleoyl-GPE (16:0/18:1)	.	-0.10	0.04	0.0148	.	-0.15	0.05	0.0038
1-palmitoyl-2-docosahexaenoyl-GPE (16:0/22:6)*	.	-0.04	0.05	0.4299	.	0.01	0.05	0.8310
1-stearoyl-2-docosahexaenoyl-GPE (18:0/22:6)*	.	0.08	0.04	0.0381	.	0.43	0.04	<.0001
1-palmitoyl-2-linoleoyl-GPE (16:0/18:2)	.	-0.06	0.05	0.1826	.	0.08	0.05	0.0967
1-stearoyl-2-linoleoyl-GPE (18:0/18:2)*	.	-0.01	0.03	0.8489	.	0.27	0.04	<.0001
1-oleoyl-2-linoleoyl-GPE (18:1/18:2)*	.	0.15	0.06	0.0148	.	0.58	0.05	<.0001
**Phospholipids - Phosphatidylinositol (PI)**	<.0001	.	.	.	<.0001	.	.	.
1-palmitoyl-2-arachidonoyl-GPI (16:0/20:4)*	.	-0.11	0.03	0.0012	.	-0.41	0.04	<.0001
1-stearoyl-2-arachidonoyl-GPI (18:0/20:4)	.	0.00	0.03	0.9639	.	-0.16	0.03	<.0001
1-stearoyl-2-oleoyl-GPI (18:0/18:1)*	.	0.10	0.05	0.0370	.	-0.55	0.07	<.0001
1-palmitoyl-2-oleoyl-GPI (16:0/18:1)*	.	-0.04	0.05	0.4867	.	-0.68	0.10	<.0001
1-palmitoyl-2-linoleoyl-GPI (16:0/18:2)	.	-0.09	0.03	0.0039	.	-0.17	0.04	<.0001
1-stearoyl-2-linoleoyl-GPI (18:0/18:2)	.	0.08	0.03	0.0138	.	-0.03	0.04	0.3836
**Sphingolipids - Ceramides**	<.0001	.	.	.	<.0001	.	.	.
ceramide (d16:1/24:1, d18:1/22:1)*	.	-0.21	0.11	0.0700	.	-0.41	0.11	0.0009
ceramide (d18:1/17:0, d17:1/18:0)*	.	-0.05	0.07	0.4112	.	-0.43	0.08	<.0001
ceramide (d18:2/24:1, d18:1/24:2)*	.	-0.03	0.05	0.5219	.	-0.23	0.06	0.0004
N-palmitoyl-sphinganine (d18:0/16:0)	.	0.16	0.08	0.0551	.	-0.37	0.12	0.0039
N-palmitoyl-sphingosine (d18:1/16:0)	.	0.06	0.03	0.0640	.	-0.22	0.05	<.0001
N-stearoyl-sphingosine (d18:1/18:0)*	.	-0.02	0.04	0.6572	.	-0.71	0.08	<.0001
N-palmitoyl-sphingadienine (d18:2/16:0)*	.	0.06	0.07	0.4030	.	-0.21	0.08	0.0114
N-stearoyl-sphingadienine (d18:2/18:0)*	.	-0.10	0.08	0.2427	.	-0.86	0.12	<.0001
N-behenoyl-sphingadienine (d18:2/22:0)*	.	-0.03	0.07	0.6469	.	-0.15	0.07	0.0469

**Sphingolipids - Dihydroceramides**	0.1211	.	.	.	0.0096	.	.	.
N-palmitoyl-sphinganine (d18:0/16:0)	.	0.16	0.08	0.0551	.	-0.37	0.12	0.0039
N-arachidoyl-sphingosine (d18:1/20:0)*	.	0.04	0.04	0.3222	.	-0.08	0.05	0.1012
**Sphingolipids - Dihydrosphingomyelins**	0.0006	.	.	.	<.0001	.	.	.
sphingomyelin (d18:0/18:0, d19:0/17:0)*	.	-0.10	0.04	0.0220	.	-0.83	0.10	<.0001
sphingomyelin (d18:0/20:0, d16:0/22:0)*	.	-0.13	0.04	0.0052	.	-0.32	0.08	0.0003
myristoyl dihydrosphingomyelin (d18:0/14:0)*	.	-0.19	0.04	<.0001	.	-0.02	0.04	0.6620
palmitoyl dihydrosphingomyelin (d18:0/16:0)*	.	-0.06	0.02	0.0085	.	-0.34	0.04	<.0001
behenoyl dihydrosphingomyelin (d18:0/22:0)*	.	-0.12	0.06	0.0601	.	-0.49	0.11	<.0001
**Sphingolipids - Hexosylceramides (HCER)**	0.0003	.	.	.	<.0001	.	.	.
glycosyl ceramide (d18:1/20:0, d16:1/22:0)*	.	-0.09	0.04	0.0171	.	-0.15	0.04	0.0007
glycosyl ceramide (d18:2/24:1, d18:1/24:2)*	.	-0.27	0.07	0.0002	.	-0.31	0.06	<.0001
glycosyl-N-palmitoyl-sphingosine (d18:1/16:0)	.	-0.07	0.03	0.0080	.	-0.20	0.03	<.0001
glycosyl-N-stearoyl-sphingosine (d18:1/18:0)	.	-0.10	0.03	0.0045	.	-0.40	0.04	<.0001
glycosyl-N-(2-hydroxynervonoyl)-sphingosine (d18:1/24:1(2OH))*	.	-0.15	0.03	<.0001	.	-0.32	0.04	<.0001
**Sphingolipids - Lactosylceramides (LCER)**	0.2302	.	.	.	<.0001	.	.	.
lactosyl-N-palmitoyl-sphingosine (d18:1/16:0)	.	-0.03	0.03	0.3573	.	0.00	0.04	0.9203
lactosyl-N-stearoyl-sphingosine (d18:1/18:0)*	.	0.03	0.03	0.4387	.	-0.29	0.06	<.0001
**Sphingolipids - Sphingosines**	0.0687	.	.	.	0.2188	.	.	.
sphingosine 1-phosphate	.	-0.18	0.10	0.0807	.	-0.12	0.10	0.2188
**Sphingomyelins**	0.0001	.	.	.	<.0001	.	.	.
sphingomyelin (d17:1/16:0, d18:1/15:0, d16:1/17:0)*	.	-0.09	0.03	0.0042	.	-0.14	0.03	0.0001
sphingomyelin (d18:1/17:0, d17:1/18:0, d19:1/16:0)	.	-0.11	0.03	0.0014	.	-0.17	0.04	<.0001
sphingomyelin (d18:1/20:2, d18:2/20:1, d16:1/22:2)*	.	-0.03	0.06	0.6095	.	-0.03	0.07	0.6379
sphingomyelin (d18:1/21:0, d17:1/22:0, d16:1/23:0)*	.	-0.09	0.03	0.0146	.	-0.07	0.04	0.0900
sphingomyelin (d18:1/22:1, d18:2/22:0, d16:1/24:1)*	.	-0.07	0.03	0.0140	.	-0.05	0.03	0.1211
sphingomyelin (d18:1/22:2, d18:2/22:1, d16:1/24:2)*	.	-0.13	0.03	0.0001	.	0.13	0.03	<.0001
sphingomyelin (d18:2/23:0, d18:1/23:1, d17:1/24:1)*	.	-0.10	0.03	0.0032	.	-0.14	0.04	0.0005
sphingomyelin (d18:1/25:0, d19:0/24:1, d20:1/23:0, d19:1/24:0)*	.	-0.07	0.05	0.1299	.	-0.21	0.06	0.0028
palmitoyl sphingomyelin (d18:1/16:0)	.	-0.06	0.02	0.0031	.	-0.20	0.02	<.0001
stearoyl sphingomyelin (d18:1/18:0)	.	-0.09	0.02	0.0001	.	-0.28	0.03	<.0001
behenoyl sphingomyelin (d18:1/22:0)*	.	-0.06	0.03	0.0494	.	-0.04	0.03	0.2687
tricosanoyl sphingomyelin (d18:1/23:0)*	.	-0.07	0.03	0.0253	.	-0.09	0.04	0.0202
lignoceroyl sphingomyelin (d18:1/24:0)	.	-0.04	0.03	0.2008	.	-0.02	0.03	0.4873
sphingomyelin (d17:2/16:0, d18:2/15:0)*	.	-0.21	0.05	<.0001	.	-0.28	0.05	<.0001
sphingomyelin (d18:1/14:0, d16:1/16:0)*	.	-0.16	0.03	<.0001	.	0.09	0.03	0.0079
sphingomyelin (d18:1/18:1, d18:2/18:0)	.	-0.10	0.02	0.0001	.	-0.39	0.03	<.0001
sphingomyelin (d18:1/19:0, d19:1/18:0)*	.	-0.08	0.03	0.0109	.	-0.06	0.03	0.0646
sphingomyelin (d18:1/20:0, d16:1/22:0)*	.	-0.06	0.02	0.0182	.	-0.01	0.03	0.6424
sphingomyelin (d18:1/20:1, d18:2/20:0)*	.	-0.09	0.03	0.0016	.	0.02	0.03	0.5114
sphingomyelin (d18:1/24:1, d18:2/24:0)*	.	-0.13	0.04	0.0020	.	-0.24	0.05	<.0001
sphingomyelin (d18:2/14:0, d18:1/14:1)*	.	-0.34	0.06	<.0001	.	0.16	0.04	0.0006
sphingomyelin (d18:2/16:0, d18:1/16:1)*	.	-0.07	0.03	0.0117	.	-0.23	0.03	<.0001
sphingomyelin (d18:2/21:0, d16:2/23:0)*	.	-0.06	0.04	0.1019	.	-0.05	0.04	0.2178
sphingomyelin (d18:2/24:1, d18:1/24:2)*	.	-0.10	0.03	0.0003	.	-0.12	0.03	0.0001
sphingomyelin (d18:2/18:1)*	.	0.03	0.03	0.3447	.	-0.23	0.04	<.0001
sphingomyelin (d18:2/23:1)*	.	-0.08	0.03	0.0155	.	-0.04	0.03	0.2073
sphingomyelin (d18:2/24:2)*	.	-0.09	0.03	0.0096	.	0.12	0.03	0.0007
**Tocopherol Metabolism**	<.0001	.	.	.	<.0001	.	.	.
alpha-CEHC	.	-0.52	0.15	0.0010	.	-0.38	0.20	0.0631
alpha-CEHC glucuronide*	.	-0.56	0.10	<.0001	.	-0.37	0.08	<.0001

alpha-CEHC sulfate	.	-0.29	0.07	0.0003	.	-0.29	0.09	0.0039
alpha-tocopherol	.	-0.08	0.01	<.0001	.	-0.12	0.01	<.0001
gamma-tocopherol/beta-tocopherol	.	-0.04	0.05	0.4058	.	-0.31	0.09	0.0020
**Tryptophan Indole Pathway**	0.0004	.	.	.	<.0001	.	.	.
2-oxindole-3-acetate	.	0.28	0.56	0.6172	.	0.67	0.74	0.3664
3-indoxyl sulfate	.	-0.21	0.07	0.0064	.	-0.18	0.05	0.0013
5-hydroxyindole sulfate	.	-0.12	0.05	0.0150	.	-0.51	0.07	<.0001
7-hydroxyindole sulfate	.	-0.10	0.04	0.0320	.	-0.19	0.06	0.0035
indoleacetate	.	0.04	0.23	0.8767	.	0.39	0.30	0.1924
indoleacetylalanine	.	-0.63	0.21	0.0050	.	0.48	0.10	<.0001
indoleacetylglutamine	.	0.06	0.26	0.8350	.	0.59	0.14	0.0001
indoleacetylglycine	.	-0.14	0.11	0.2204	.	0.23	0.14	0.1048
indoleacrylate	.	0.01	0.15	0.9679	.	0.19	0.16	0.2227
indolelactate	.	-0.20	0.07	0.0055	.	0.02	0.18	0.8931
indolepropionate	.	-0.05	0.18	0.7645	.	0.45	0.11	0.0003
indolin-2-one	.	-0.20	0.08	0.0201	.	-0.20	0.06	0.0042
indoxyl glucuronide	.	-0.12	0.16	0.4650	.	0.31	0.12	0.0135

Supplementary Material 7: PICRUSt-predicted functional analysis for fecal tryptophan pathway

Green and red indicate the enzyme function significantly increased or decreased, respectively, in feces of dogs fed TF1 vs CF.

KO_ID	Enzyme name	Pob |t|
K00128	aldehyde dehydrogenase (NAD+) [EC:1.2.1.3]	0.1543
K00164	2-oxoglutarate dehydrogenase E1 component [EC:1.2.4.2]	<.0001
K00252	glutaryl-CoA dehydrogenase [EC:1.3.8.6]	0.9803
K00274	monoamine oxidase [EC:1.4.3.4]	0.222
K00452	3-hydroxyanthranilate 3,4-dioxygenase [EC:1.13.11.6]	<.0001
K00453	tryptophan 2,3-dioxygenase [EC:1.13.11.11]	0.0795
K00466	tryptophan 2-monooxygenase [EC:1.13.12.3]	0.0001
K00486	kynurenine 3-monooxygenase [EC:1.14.13.9]	<.0001
K00543	acetylserotonin N-methyltransferase [EC:2.1.1.4]	0.7738
K00626	acetyl-CoA C-acetyltransferase [EC:2.3.1.9]	0.7507
K01426	amidase [EC:3.5.1.4]	0.0075
K01501	nitrilase [EC:3.5.5.1]	0.0578
K01556	kynureninase [EC:3.7.1.3]	0.0101
K01593	aromatic-L-amino-acid / L-tryptophan decarboxylase [EC:4.1.1.105 / 4.1.1.28]	0.5309
K01667	tryptophanase [EC:4.1.99.1]	<.0001
K01692	enoyl-CoA hydratase [EC:4.2.1.17]	0.043
K01721	nitrile hydratase subunit alpha [EC:4.2.1.84]	0.0012
K01782	3-hydroxyacyl-CoA dehydrogenase / enoyl-CoA hydratase / 3-hydroxybutyryl-CoA epimerase [EC:5.1.2.3 / 4.2.1.17 / 1.1.1.35]	0.0002
K01825	3-hydroxyacyl-CoA dehydrogenase / enoyl-CoA hydratase / 3-hydroxybutyryl-CoA epimerase / enoyl-CoA isomerase [EC:5.3.3.8 / 5.1.2.3 / 4.2.1.17 / 1.1.1.35]	0.0013
K03392	aminocarboxymuconate-semialdehyde decarboxylase [EC:4.1.1.45]	0.0001
K03781	catalase [EC:1.11.1.6]	0.0571
K03782	catalase-peroxidase [EC:1.11.1.21]	0.0004
K04103	indolepyruvate decarboxylase [EC:4.1.1.74]	0.261
K07130	arylformamidase [EC:3.5.1.9]	0.0163
K10217	aminomuconate-semialdehyde / 2-hydroxymuconate-6-semialdehyde dehydrogenase [EC:1.2.1.85 / 1.2.1.	0.0909
K14338	cytochrome P450 / NADPH-cytochrome P450 reductase [EC:1.6.2.4 / 1.14.14.1]	0.2709
